# Selection and validation of reference genes for quantitative real-time PCR in the green microalgae *Tetraselmis chui*

**DOI:** 10.1371/journal.pone.0245495

**Published:** 2021-01-14

**Authors:** Sonia Torres, Carmen Lama, Lalia Mantecón, Emmanouil Flemetakis, Carlos Infante

**Affiliations:** 1 Fitoplancton Marino, S.L., El Puerto de Santa María, Cádiz, Spain; 2 Laboratory of Molecular Biology, Department of Biotechnology, Agricultural University of Athens, Athens, Greece; University of New England, AUSTRALIA

## Abstract

Quantitative real-time reverse transcription PCR (RT-qPCR) is a highly sensitive technique that can be applied to analyze how genes are modulated by culture conditions, but identification of appropriate reference genes for normalization is a critical factor to be considered. For this reason, the expression stability of 18 candidate reference genes was evaluated for the green microalgae *Tetraselmis chui* using the widely employed algorithms geNorm, NormFinder, BestKeeper, the comparative Δ*C*_T_ method, and RefFinder. Microalgae samples were collected from large scale outdoor photobioreactors during the growing phase (OUT_GP), and during the semi-continuous phase at different times of the day (OUT_DC). Samples from standard indoor cultures under highly controlled conditions (IND) were also collected to complement the other data. Different rankings for the candidate reference genes were obtained depending on the culture conditions and the algorithm employed. After comparison of the achieved ranks with the different methods, the references genes selected for samples from specific culture conditions were *ALD* and *EFL* in OUT_GP, *RPL32* and *UBCE* in OUT_DC, and *cdkA* and *UBCE* in IND. Moreover, the genes *EFL* and *cdkA* or *EFL* and *UBCE* appeared as appropriate combinations for pools generated from all samples (ALL). Examination in the OUT_DC cultures of genes encoding the large and small subunits of ADP-glucose pyrophosphorylase (*AGPL* and *AGPS*, respectively) confirmed the reliability of the identified reference genes, *RPL32* and *UBCE*. The present study represents a useful contribution for studies of gene expression in *T*. *chui*, and also represents the first step to set-up an RT-qPCR platform for quality control of *T*. *chui* biomass production in industrial facilities.

## Introduction

Microalgae represent the most phylogenetically diverse group of organisms on Earth [1,2]. As photosynthetic organisms, they can utilize solar energy to convert inorganic CO_2_ into a wide set of valuable biologically active compounds, including fatty acids, pigments, vitamins, and proteins and enzymes. Owing to the metabolic plasticity of microalgae, stress caused by changes in factors such as salinity, temperature, light and nutrients, can lead to the production and accumulation of unique metabolites [3,4]. Such molecules have neurological, antimicrobial, antifungal, antiprotozoal, antiviral, anti-inflammatory, antioxidant, anticancer and immune-stimulant activities, and this explains the high potential of microalgae for cosmetics, pharmaceuticals, feed and food, and as a source of and supplements for nutraceuticals [5–7].

The green algae genus *Tetraselmis* (Chlorodendrophyceae) comprises species that are among the most widely used in aquaculture feed for larval nutrition in molluscs, shrimp and fish, as well as for the enrichment of rotifers and *Artemia* nauplii [8,9]. More specifically, *Tetraselmis chui* (Butcher 1959) exhibits an appropriate content of proteins, lipids, carbohydrates and essential fatty acids and sterols, for the maintenance of aquaculture organisms [10,11]. In addition to its nutritional value, some studies suggest that *T*. *chui* supplementation can enhance the immune system and health in the organisms to which it is fed. For example, inclusion of *T*. *chui* in formulated feed enhances defense against oxidative stress and damages in Pacific white-leg shrimp (*Litopenaeus vannamei)* postlarvae [12], and improves immune-related parameters in the fish, gilthead seabream (*Sparus aurata*) [13,14]. Furthermore, the company Fitoplancton Marino, S.L. (El Puerto de Santa María, Cádiz, Spain) has been authorized since 2014 to market its lyophilized *T*. *chui* biomass as a novel food for human consumption in the European Union (EU) according to Regulation (EC) No 258/97. In 2017, commercialization by Fitoplancton Marino, S.L. of the lyophilized biomass, TetraSOD^®^, for use as a food supplement for humans was also approved in the EU [15].

It has been stated that gene expression analysis by quantitative real-time reverse transcription polymerase chain reaction (RT-qPCR) represents an important tool for understanding biological and molecular mechanisms underlying cellular processes [16–18]. The comparison of the gene expression patterns across different experimental or biological conditions can provide valuable insights into the regulation of the metabolic pathways that help cells withstand or adapt to environmental change. In this context, RT-qPCR is one of the most commonly used approaches to measure gene expression levels owing to its accuracy, sensitivity, specificity, precision, real-time detection of the reaction progress, dynamic range, and the speed of analysis [19–22]. However, a range of factors can significantly diminish the accuracy and reliability of RT-qPCR data, such as quality and quantity of the mRNA templates, enzymatic efficiency of the reverse transcription and PCR amplification reaction, and differences between cells or tissues in the overall transcriptional activity and mRNA half-life [23,24]. To avoid the influence of these factors, they are usually controlled by normalizing gene expression to an internal control or reference gene. Such reference genes should have an expression unaffected by experimental factors, have minimal variation in expression between tissues and physiological states of the organism, and a threshold cycle similar to the target gene. However, studies have revealed that it is difficult to identify universal reference genes that do not vary under different experimental conditions or with cell type or organism, and hence it is essential to take this into account when identifying reference genes in order to ensure the accuracy of RT-qPCR analysis [24–26]. Several statistical algorithms have been developed to identify the most suitable reference genes under a given set of experimental or biological conditions in order to avoid artifacts due to normalization, and include geNorm [27], NormFinder [28], BestKeeper [29], the comparative Δ*C*_T_ method [30], and the web-based program RefFinder [31]. These tools have been carried out on a broad range of species, including whole organisms, specific tissues or cell types from animals [19–21,32,33], plants [34–37], and fungi [38,39]. Selection of reference genes has also been addressed in macroalgae [40–42] as well as in specific microalgae species, including the diatoms *Phaeodactylum tricornutum* [43], *Pseudo-nitzschia multristrata* and *P*. *arenysensis* [44], *Ditylum brightwellii* [45] and *Skeletonema marinoi* [46], the dinoflagellates *Prorocentrum minimum* [47], *Symbiodinium* [48], *Alexandrium catanella* [49] and *Karenia mikimotoi* [50], the haptophyte *Isochrysis zhangjiangensis* [51], the eustigmatophyte *Nannochloropsis* [52,53], the raphidophyte *Heterosigma akashiwo* [54], or the green algae *Volvox carteri* [55], *Closterium ehrenbergii*, [56], *Chlamydomonas* [17,57], and *Tetraselmis suecica* [58]. However, to our knowledge, there has been no study validating reference genes for the green microalgae species *T*. *chui*.

In the present study, the suitability of a set of 18 different candidate reference genes for normalization in RT-qPCR assays in the microalgae species *T*. *chui* were evaluated. The candidate genes were chosen based on their previous assessment in other organisms, particularly in other microalgae. The candidate genes included, 18S rRNA (*18S*), actin (*ACT*), aldolase A (*ALD*), alpha tubulin (*aTUB-1* and *aTUB-2*), beta tubulin (*bTUB*), cyclin dependent kinase A (*cdkA*), elongation factor-like (*EFL*), glyceraldehyde-3-phosphate dehydrogenase (*GAPDH*), histone 2A (*His2A*), beta-ketoacyl-ACP synthase (*KAS*), large subunit of the ribulose-1,5-biphosphate carboxylase (*rbcL*), ubiquitin conjugating enzyme (*UBCE*), phosphoglycerate kinase (*PGK*), ribosomal protein S10 (*RPS10*) and L32 (*RPL32*), and eukaryotic translation initiation factor 2 (*eIF2-1* and *eIF2-2*). *T*. *chui* microalgae samples were first collected from outdoor photobioreactors dedicated to the industrial production of biomass operated in semi-continuous mode. Sampling was performed from initial inoculation in photobioreactors until the cultures reached the appropriate cell density for daily harvesting and spanned 18 days. Thereafter, samples were drawn from outdoor photobioreactors at five different hours of the diurnal cycle from dawn to dusk. The collected samples represented microalgae exposed to strong differences in light irradiance (ranging from 3 to 1300 μmol photons m^-2^ s^-1^ of photosynthetically active radiation (PAR)) and also in culture temperature (ranging from 10.6 to 22.8°C). Further samples were obtained from indoor cultures grown under stable temperature and light irradiance and spanned the growth curve from the initial inoculation to the stationary phase. Thereafter, the transcript abundance of the candidate reference genes were determined by RT-qPCR, and analyzed using geNorm, NormFinder, BestKeeper, the comparative Δ*C*_T_ method, and RefFinder to identify reference genes suitable for gene expression normalization. Finally, the expression of the genes encoding for the large and small subunits of ADP-glucose pyrophosphorylase (*AGPL* and *AGPS*, respectively) were examined by using the most stable reference genes selected.

## Materials and methods

### Strain and culture conditions

*T*. *chui* strain CCFM-03 (belonging to the Culture Collection of Fitoplancton Marino, S.L.) is cultured for industrial production of biomass in the facilities of the company Fitoplancton Marino, S.L. For the purpose of this study, 4000 L closed tubular outdoor photobioreactors were sampled. Microalgae cells were grown in seawater with f/2 culture medium [59] throughout the production cycle, including the first indoor stages during culture up-scaling through to outdoor cultures. The composition of f/2 medium was: 8.82 x 10^−4^ M NaNO_3_, 3.62 x 10^−5^ M NaH_2_PO_4_·H_2_O, 1.06 x 10^−4^ M Na_2_CO_3_, 1.17 x 10^−5^ M FeCl_3_·6(H_2_O), 1.17 x 10^−5^ M Na2(EDTA)·2(H_2_O), 3.93 x 10^−8^ M CuSO_4_·5(H_2_O), 2.6 x 10^−8^ M Na_2_MoO_4_·2(H_2_O), 7.65 x 10^−8^ M ZnSO_4_·7(H_2_O), 4.2 x 10^−8^ M CoCl_2_·6(H_2_O), 9.1 x 10^−7^ M MnCl_2_·4(H_2_O), 2.96 x 10^−7^ M vitamin B1, 2.05 x 10^−9^ M vitamin H, and 3.69 x 10^−10^ M vitamin B12. In outdoor cultures, the microalgae were exposed daily to ambient conditions, with temperature and light intensity being probably the most significant parameters that changed (S1 Table). Culture conditions that were strictly controlled included pH (maintained at 7.5–8.5 by on-demand CO_2_ injections) and nitrate content, which was maintained over 500 μM. Indoor cultures of microalgae were maintained under controlled conditions at 22°C in a thermoregulated room with a photoperiod of 24 h of cool fluorescent white light, and ~150 μmol photons m^-2^ s^-1^ of PAR. All microalgae cultures were continuously aerated using compressed air containing 2% CO_2_.

### Microalgae samples

Three different conditions were tested in the study: i) highly controlled indoor cultures (IND), ii) outdoor cultures during the growth phase (OUT_GP), that was, from initial inoculation of the photobioreactors until the cultures reached an appropriate cell density for semi-continuous cultivation, and iii) outdoor cultures during the semi-continuous phase (OUT_DC), in which the culture was maintained in exponential growth over ~2 months by daily harvesting of biomass followed by addition of nutrients to restore adequate levels of nitrate, salts and vitamins.

For the IND cultures, three 5 L flasks were inoculated on day 1 (D1) at an average initial cell density of 85 x 10^3^ cells/ml (1/20 dilution of a well grown source culture), which was determined using a haematocytometer (Neubauer counting chamber) and a microscope. Microalgae samples were withdrawn from each of the triplicate flasks for cell density determination and RNA isolation on D1, D4, D7, D10 and D13. Samples from D4 and D7 corresponded to the exponential growth phase and samples from D10 and D13 corresponded to the stationary growth phase (S1 Fig). A total of 15 samples were collected (three per time point) for gene expression analysis. Collection of samples for both cell density determination and RNA isolation occurred at 9:00 a.m. on the five sampling days.

OUT_GP samples were collected from three 4000 L photobioreactors of the same production unit after inoculation on D1 (with an average cell density of 20 x 10^3^ cells/ml), and then at D4, D7, D11, and finally at D18 (the day before the commencement of the semi-continuous cultivation mode) during the exponential growth phase. In all instances, the samples were taken at 1:00 p.m. Samples were collected for cell density determination (S2 Fig) and 15 samples (three per time point) were collected for RNA isolation and further gene expression analysis.

OUT_DC samples were collected from three 4000 L photobioreactors of the same production unit and during the semi-continuous cultivation mode. Samples were withdrawn 13 days after the commencement of the semi-continuous phase (approximately 30 days after inoculation of the photobioreactors) for RNA isolation at five different times of the diurnal cycle: 7:00 a.m., 10:00 a.m., 1:00 p.m., 4:00 p.m. and 7:00 p.m. Thus, a total of 15 samples (three per time point) were processed for gene expression analysis. The average cell density at 7:00 a.m. (the first sample of a given was 7.4 x 10^6^ cells/ml.

The temperature of cultures in OUT_GP and OUT_DC was automatically controlled with a probe at each sampling time, and PAR was also recorded using a radiometer sensor LI-250A Light Meter (LICOR Biosciences). The data recorded is presented in S1 Table.

### Total RNA isolation and cDNA synthesis

In each sample, an appropriate volume of culture was drawn to give ~4 x 10^6^ cells for RNA isolation. Cells were centrifuged at 5,000 g for 5 min, and the supernatant was removed. Microalgae cells were homogenized using stainless steel beads (0.2 mm, Next Advance) for 3 min at speed 10 in a Bullet Blender^®^ 24 (Next Advance). Total RNA was extracted using a NucleoSpin^®^ Plant II kit (Macherey-Nagel) following the manufacturer’s instructions. The extracted RNA was treated twice with DNase I to avoid further PCR amplification of residual traces of genomic DNA. RNA samples were quantified using a NanoDrop 2000 spectrophotometer (Thermo Scientific) and the quality was checked in agarose gels.

Total RNA (1 μg) from each sample was reverse-transcribed using an iScript™ cDNA Synthesis kit (Bio-Rad) in a reaction volume of 20 μL according to the manufacturer’s protocol. All cDNA reactions were finally diluted 10-fold by adding 180 μL of nuclease-free water. The absence of genomic DNA contamination was confirmed by direct PCR amplification of two randomly selected RNA samples in the absence of cDNA synthesis.

### Selection of candidate reference genes for RT-qPCR

An initial set of 16 genes involved in different cellular processes were pre-selected to assess their suitability as reference genes for RT-qPCR analysis. The selection of candidate reference genes was based on previous gene expression studies of other microalgae species and other organisms. The nucleotide sequence corresponding to *18S* and *rbcL* from *T*. *chui* was directly retrieved from GenBank/EMBL/DDBJ database (S2 Table). The nucleotide sequences of the other candidate genes were identified in the available transcript sequences (~22,600) for the *T*. *chui* strain PLY429 in the iMicrobe database (www.imicrobe.us) after annotation with the AutoFACT tool [60]. EditSeq v8.1.3 (DNASTAR) was used to identify the coding sequences of candidate genes as well as the deduced protein sequences, and then gene identities were confirmed with BLASTp (S2 Table). Two different coding sequences were found in *T*. *chui* for both alpha tubulin (*aTUB-1* and *aTUB-2*) and eukaryotic translation initiation factor 2 (*eIF2-1* and *eIF2-2*), and all four genes were included in this study. The isolation of the target genes *AGPL* and *AGPS* followed the same general strategy outlined above.

### Primer design and RT-qPCR

Specific primer pairs (Table 1) for the 18 selected candidate reference genes (and also for the target genes *AGPL* and *AGPS*) were designed using Oligo *v*7.60 software (Molecular Biology Insights). To ensure maximum specificity and efficiency in PCR amplification, highly stringent conditions were initially selected for primer design. For each primer pair these were: ΔG of the most stable 3'-dimer > -3.5 Kcal/mol, ΔG of the most stable dimer overall > -7.5 Kcal/mol, no hairpins with ΔG < -1 Kcal/mol and a melting temperature (T_m_) > 40°C, primer efficiency (PE) > 480, PE of false priming sites < 100, optimal annealing temperature (T_a_) of the PCR product between 62–65°C, primer lengths of 20–28 nucleotides, and amplicon length ranging between 100–200 base pairs. When most of the criteria were successfully met, the recommended temperature for annealing/extension in PCR was 68°C. All designed primers were finally ordered from a commercial supplier (Eurofins). Appropriate performance of each primer pair was initially tested by PCR amplification of the target amplicons employing the same conditions described below for RT-qPCR. Specificity of each primer pair was verified by melting curve analysis from 70°C to 95°C with a ramp speed of 0.5°C every 10 s. Single, sharp peaks were obtained in all instances, thus ruling out amplification of non-specific products or primer dimer artifacts (S3 Fig). To determine real-time PCR efficiency (*E*) of each primer pair, standard curves were prepared from serial dilutions of cDNA (from 100 ng to 0.01 ng) and the results plotted in Excel against their *C*_T_ values (S4 Fig). Then, slopes of the linear regressions were determined applying the equation *E* = 10^−1/slope^ as previously reported [61]. The linear correlation coefficient (R^2^) of each primer pair was also determined in Excel. The PCR products were analyzed by standard agarose gel electrophoresis (2.5% w/v in TAE 1X) and clear DNA bands of the expected sizes were obtained (S5 Fig).

**Table 1:** List of primers used for RT-qPCR.

CANDIDATE REFERENCE GENES						
Gene symbol	Primer sequence	Amplicon length (bp)	Optimal T_a_ (°C)^a^	PCR efficiency (*E*)		Correlation coefficient (R^2^)	
*ACT*	F: 5’-AGAAGACCTATGAGCTGCCCGACG-3’	168	64.0	1.06		1.00	
	R: 5’-GGTCCTTACGGATATCGACATCGCACT-3’						
*EFL*	F: 5’-CCGGCGAGATCAAGGTCGGCTAC-3’	171	63.9	1.05		1.00	
	R: 5’-AGGGCTTGAAGACAATGGTGGATACCTC-3’						
*His2A*	F: 5’-TCATCGTTTGCTGAAGAACCGTGTGAC-3’	146	62.3	1.06		1.00	
	R: 5’-TGCGCTTCACCTTCAGATCCTTAGACG-3’						
*ALD*	F: 5’-TGTACAAGCCCGGCAACGTGA-3’	176	64.9	1.03		0.99	
	R: 5’-CTTGATGACGCCGTAGCCGAT-3’						
*cdkA*	F: 5’-ACCGCAGAACTTACTGATTGACCGT-3’	123	61.2	1.07		1.00	
	R: 5’-CGGTACCACAGAGTCACAACCTCGT-3’						
*GAPDH*	F: 5’-GCCATCGCGCTCATCTACCCCGAA-3’	110	62.4	1.07		0.99	
	R: 5’-TTCCGCTTCACTTCAAAGACACAATCGGT-3’						
*KAS*	F: 5’-CGATGCACACCACATGACCGACC-3’	172	65.5	0.95		1.00	
	R: 5’-CGCCTGCTTCATCGCCTTGACC-3’						
*rbcL*	F: 5’-ACGTAAATTCACAAGCTTTCATGCGTTGG-3’	140	57.0	1.05		1.00	
	R: 5’-CATCATTTCTTCACAAGTCCCAGCCGTT-3’						
*RPL32*	F: 5’-CTCCAACAAGAAGACCCGCCACC-3’	131	62.3	1.02		1.00	
	R: 5’-TGGACACGTTCTTGGCGACCTC-3’						
*aTUB-1*	F: 5’-ACTCGACTGCTATCGCTGAAGTCTTCTCA-3’	118	62.0	0.99		1.00	
	R: 5’-GAGAACTCGCCCTCCTCCATACCCTCA-3’						
*aTUB-2*	F: 5’-CCCTCGCGCTGTGTTCATCGAC-3’	109	62.9	1.01		0.99	
	R: 5’-GTCTTCCTTTCCGGAGATCAGCTGCTCA-3’						
*bTUB*	F: 5’-GCCAGATCTTCCGCCCTGACAAC-3’	133	63.0	1.02		1.00	
	R: 5’-GACTCGGCCTCCTTACGGACCAC-3’						
*18S*	F: 5’-GGGGGAGTATGGTCGCAAGGCTGAA-3’	183	62.4	0.96		1.00	
	R: 5’-AACTAAGAACGGCCATGCACCACCAC-3’						
*UBCE*	F: 5’-CCAAACATCAACAGCAACGGCAGCA-3’	149	64.1	0.98		1.00	
	R: 5’-TGCGCAATCTCGGGCACCAG-3’						
*PGK*	F: 5’-CAACAAGTGCGACAAGATCATCATTGGC-3’	178	63.7	1.01		1.00	
	R: 5’-AACGACGACATCGGTAGGGAGCAA-3’						
*RPS10*	F: 5’-CAAGAAGAACCGCCGCGAGGTGT-3’	172	63.0	1.02		1.00	
	R: 5’-CCACGCAAAACGCTCAGTGACCAG-3’						
*eIF2-1*	F: 5’-CCTTTGGAAAGATTCGAGCAATGACGGAC-3’	129	65.1	1.04		1.00	
	R: 5’-CCGCCACCGTGAACTCGTCT-3’						
*eIF2-2*	F: 5’-CTTTGATCGTGAACCGTGGTGTGCT-3’	154	63.2	1.00		0.99	
	R: 5’-ACACCCTTGATTCCAGCCACTACGAC-3’						
TARGET GENES						
Gene symbol	Primer sequence	Amplicon length (bp)	Optimal T_a_ (°C)	PCR efficiency (*E*)		Correlation coefficient (R^2^)	
*AGPL*	F: 5’-CCGCCACCATCACTCCCGAAT-3’	164	63.2	1.06		1.00	
	R: 5’-CCGCACGAGGTCCTGATAGTCCAT-3’						
*AGPS*	F: 5’-GACTTTCTCATCCTCTCCGGCGACCA-3’	163	66.5	0.96		1.00	
	R: 5’-TCCGCCCGCTGTCGTCGATC-3’						

^a^ T_a_ represents annealing temperature.

RT-qPCR was conducted using a CFX96™ Real-Time PCR Detection System (Bio-Rad). Each 10 μL reaction contained 5 μL of 2X iQ™ SYBR^®^ Green Supermix (Bio-Rad), 300 nM of both forward and reverse primers (0.3 μL of a 10 μM stock each), 2 μL of cDNA (corresponding to the cDNA retrotranscribed from 10 ng of RNA), and 2.4 μL of nuclease-free water. All reactions were run in duplicate, and the mean threshold cycle (*C*_T_) of each sample (S3 Table) was used for further calculations. Relative transcript levels of the target genes were determined using the 2^-ΔΔCt^ method [62]. The thermal cycling profile included an initial incubation at 95°C for 3 min, followed by 40 cycles of 95°C for 15 s and 68°C for 30 s.

### Analysis of expression stability of the candidate reference genes

Five different approaches were employed to determine the expression stability of the candidate reference genes. The geNorm, or pairwise comparison approach, ranks candidate genes according to their expression stability [27]. The gene stability measure, or *M*, is determined for a given reference gene as the average pairwise variation for that gene in relation to the remaining tested reference genes. The gene with the highest *M* is stepwise excluded, with a new *M* value being repeatedly calculated for the remaining genes until a final pair of genes with the lowest *M* (the most stable) are obtained. Moreover, this Excel-based software determines the optimal number of references genes required for an accurate normalization of real-time PCR data. For this, the pairwise variation (*V*_n_/*V*_n+1_) was analyzed between the normalization factors NF_n_ and NF_n+1_; *V*_n_ values were calculated by stepwise inclusion of additional reference genes until the (n+1) gene did not contribute significantly to the newly determined normalization factor. NormFinder software (also Excel-based) is a model-based approach that ranks the candidate reference genes according to their minimal combined inter- and intra-group expression variation [28]. Such variations are combined to give a stability value, with the lowest value corresponding to the most stable expression. BestKeeper (an Excel-based statistical method) calculates the standard deviation (SD) and coefficient of variation (CV) from the input raw *C*_T_ values, with genes exhibiting the lowest SD and CV being considered as the most stable [29]. Reference genes with SD higher than 1 are considered as inconsistent and are excluded. The comparative Δ*C*_T_ algorithm uses the average SD values to rank the stability of all candidate reference genes, and the reference gene with the lowest SD is considered as the most stable [30]. Finally, as a complementary analysis to assess reference gene stability, the web-based tool RefFinder was employed to integrate the results from the four previous software packages, generating a comprehensive rank list of candidate genes [31].

### Statistical analysis of RT-qPCR data

Statistical analyses to determine relative gene expression variations in OUT_DC, both for *AGPL* and *AGPS*, were conducted using Prism 6 (GraphPad Software). Changes in transcript abundance were obtained after: i) normalization with the two most stable reference genes using the combined outputs of geNorm, NormFinder, BestKeeper, and the comparative Δ*C*_T_ method for the OUT_DC condition (*RPL32* and *UBCE*), ii) normalization with the two genes exhibiting the highest stability with RefFinder in OUT_DC (*RPS10* and *rbcL*), or iii) normalization with the two genes exhibiting the lowest stability with all the employed methods in OUT_DC (*PGK* and *eIF2-1*). The relative gene expression levels were also determined for *AGPL* and *AGPS* after normalization with the most stable genes found in the global analysis of all samples and conditions (ALL) based on the combined results of geNorm, NormFinder, BestKeeper, and the comparative Δ*C*_T_ method (*EFL* and *cdkA*), as well as with the combination of *EFL* (top-ranked by RefFinder) with another of the most stably-expressed genes found by RefFinder (*ACT*). In all instances, data were analyzed using the Friedman test (non-parametric one-way ANOVA), and when significant, the Dunn’s multiple comparison test was performed. Significance was accepted for *P* < 0.05.

## Results

### Expression levels of the candidate reference genes

**Fig 1:**
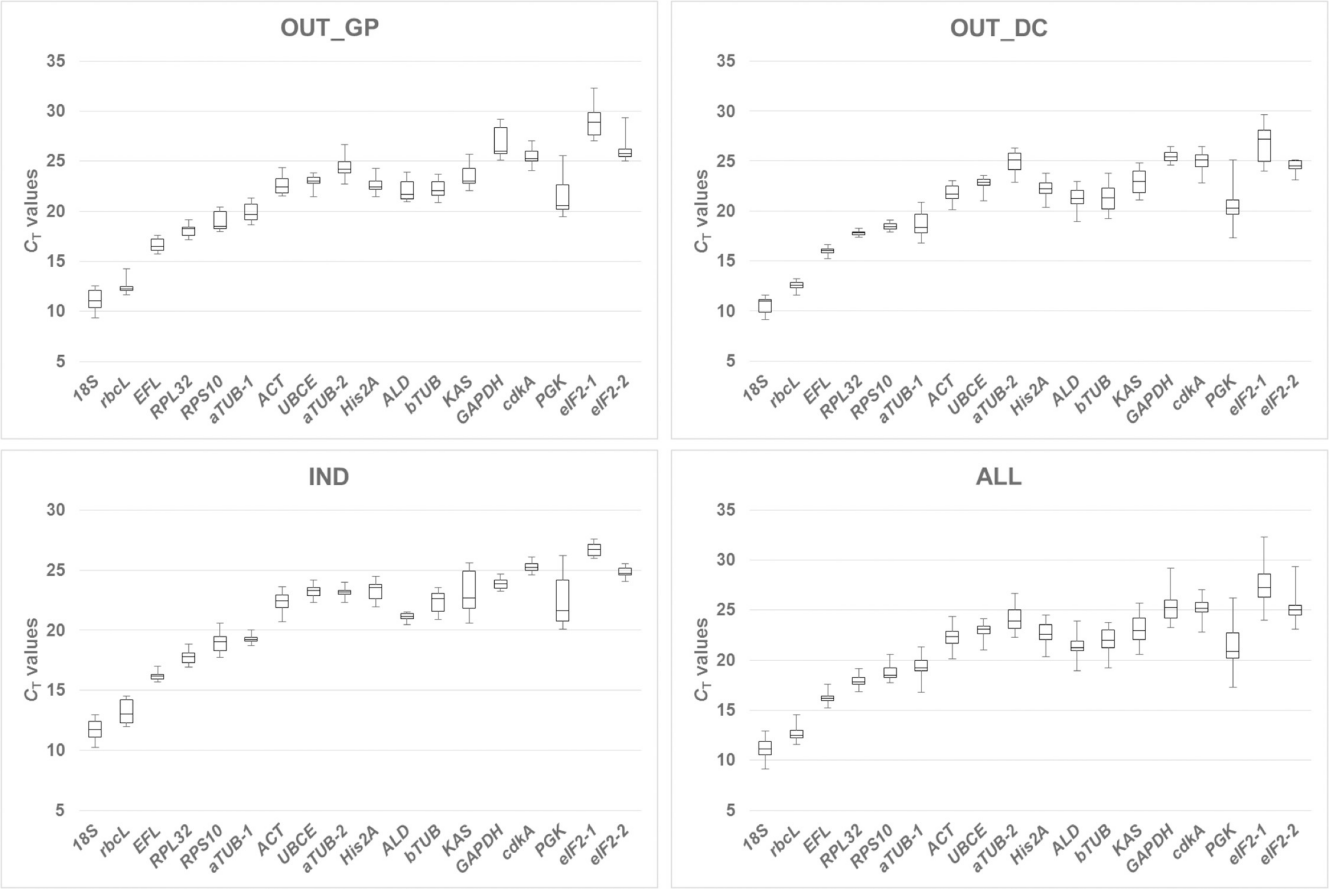
*C*_T_ values of candidate reference genes in all tested microalgae samples and conditions. The thick central line in boxes represents the median value, whereas the lower outline and the upper outline in boxes show the quartile 1 (Q1) and 3 (Q3), respectively. Whiskers are set at 1.5 times the interquartile range (IQR) both above Q3 and below Q1.

The expression stability of the candidate reference genes in *T*. *chui* was first assessed through the analysis of *C*_T_ values in real-time PCR (a high *C*_T_ means that the gene has a low expression level, and vice versa). Variation of data was compared between OUT_GP, OUT_DC and IND conditions, and with all data (ALL) being analyzed together (Fig 1). All the examined genes exhibited amplification prior to cycle n° 40 of the PCR amplification profile, with *eIF2-1* always showing the highest *C*_T_ (in some instances close to 33) across all conditions. In contrast, the lowest *C*_T_ values (and hence the highest expression levels) were observed for *18S*, and in some samples it was slightly higher than 9. The smallest variation of *C*_T_ values in OUT_GP corresponded to *EFL* and it ranged from 15.73 to 17.58, a difference of 1.85. In OUT_DC and IND, *RPL32* (from 17.37 to 18.27, and a difference of 0.90) and *ALD* (from 20.48 to 21.54, and a difference of 1.06), respectively had the smallest variation. In the global analysis ALL, the lowest range of *C*_T_ values was 16.90 to 19.13 and corresponded to *RPL32*. In contrast, the highest range of *C*_T_ values in each of the three conditions tested, and also in ALL, was observed for *PGK*; 6.06 for OUT_GP, 7.80 for OUT_DC, 6.12 for IND and 8.92 for ALL. High variations in *C*_T_ values across samples and conditions indicate unsuitable reference genes. In the present study all 18 candidate reference genes were analyzed further.

### Expression stability of reference genes

#### geNorm analysis

**Fig 2:**
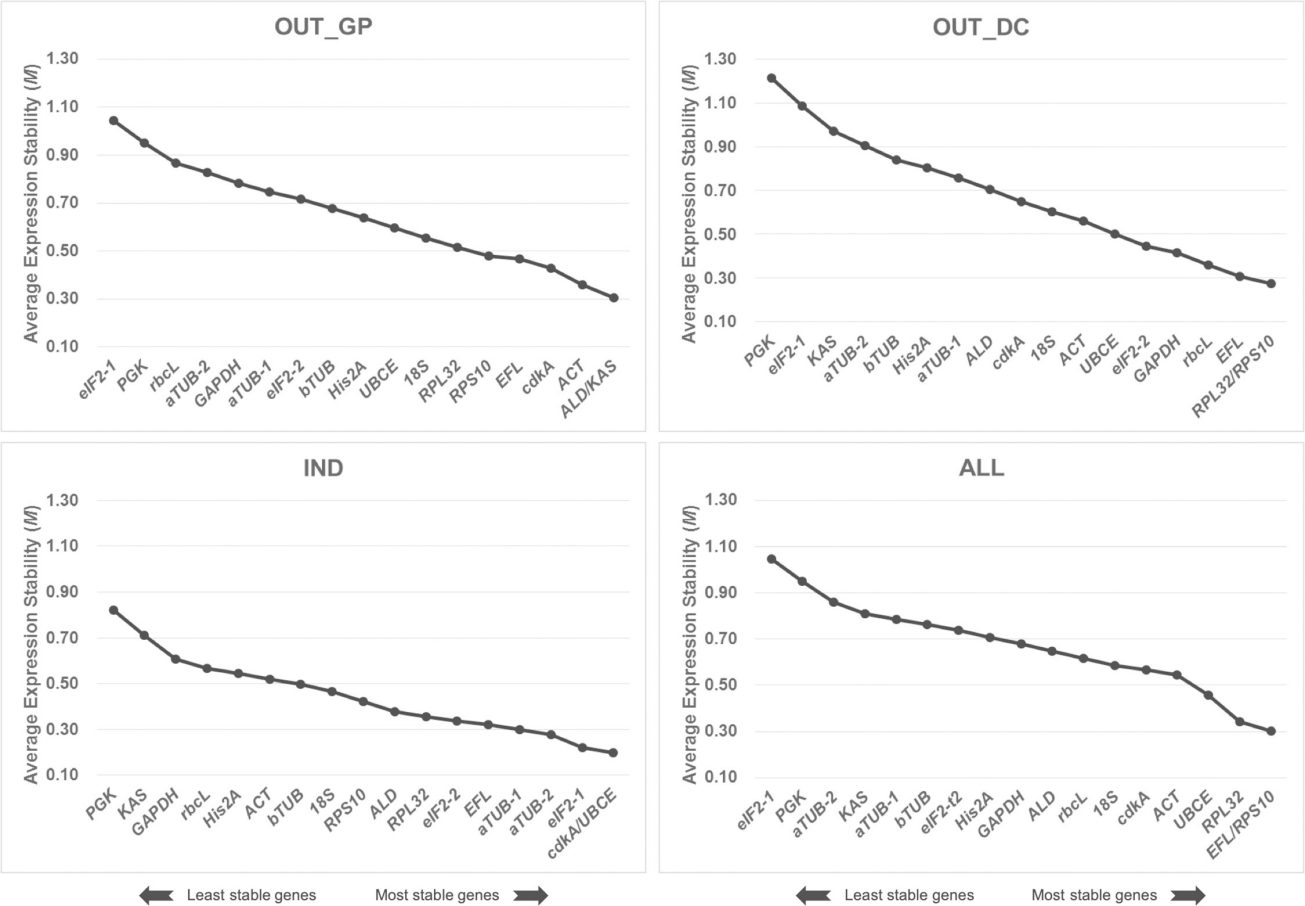
Global ranking of the 18 candidate reference genes using geNorm analysis. Average expression stability (*M*) was calculated by stepwise exclusion of the least stable gene identified by geNorm software in all tested samples (a total of 45) and conditions (OUT_GP: Outdoor cultures during the growth phase, OUT_DC: Outdoor cultures during the semi-continuous phase, IND: Indoor cultures, ALL: Combination of the three previous conditions). A lower *M* value indicates a more stable expression pattern.

**Fig 3:**
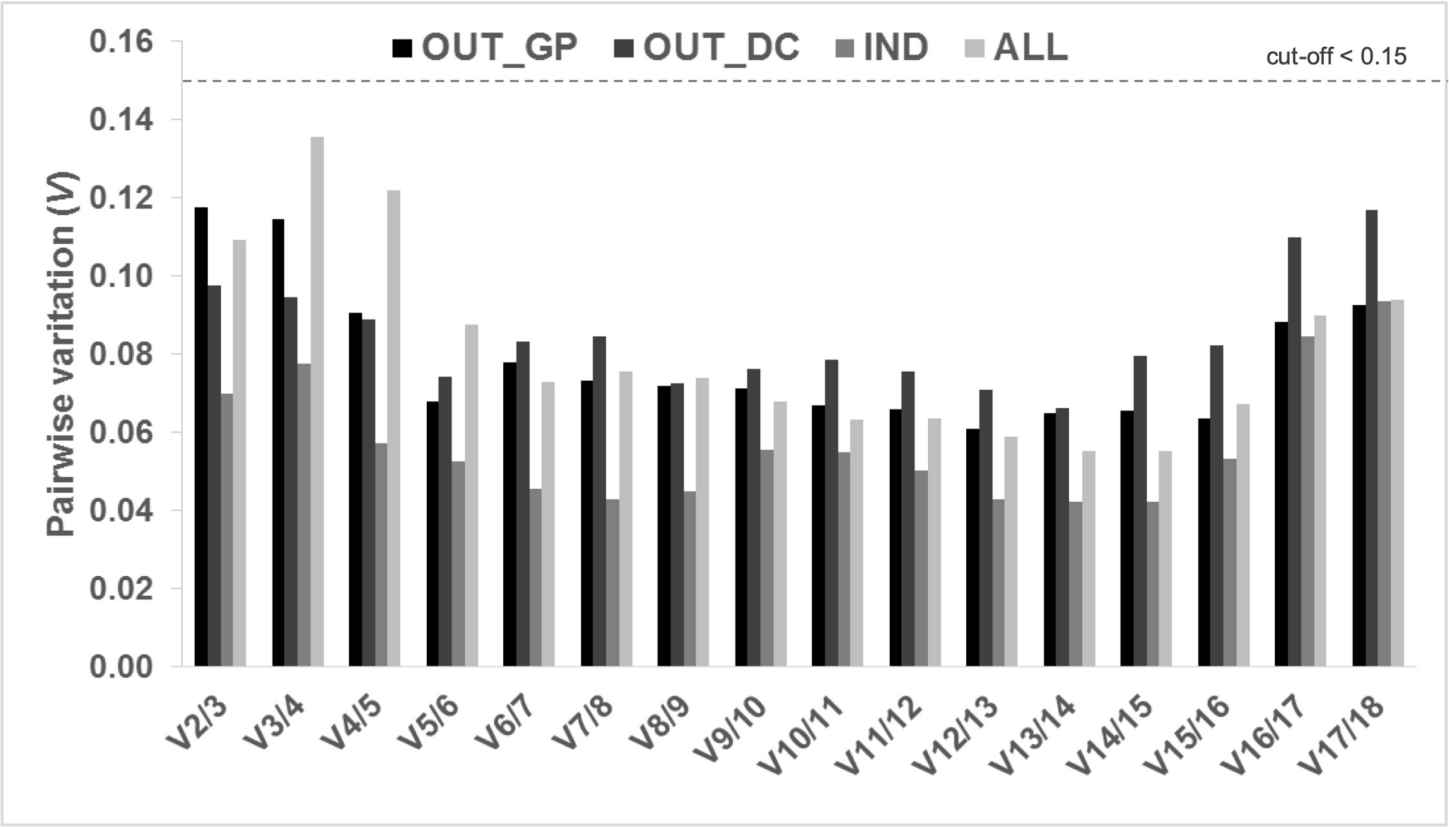
Determination of the optimal number of reference genes for accurate normalization of gene expression analysis. Pairwise variation was calculated in all tested samples (a total of 45) and conditions (OUT_GP: Outdoor cultures during the growth phase, OUT_DC: Outdoor cultures during the semi-continuous phase, IND: indoor cultures, ALL: Combination of the three previous conditions). The pairwise variation (*V*_n_/*V*_n+1_) was analyzed between the normalization factors NF_n_ and NF_n+1_.

As a starting point, the average expression stability (*M*) values of the 18 candidate reference genes were determined using the geNorm algorithm (Fig 2). Lower *M* values indicate higher stability. In all instances, candidate genes included in this study displayed an *M* value below the geNorm threshold of 1.5, thus revealing all of them as suitable for gene expression normalization. The genes with the most stable expression differed with condition and *ALD* and *KAS*, *RPL32* and *RPS10*, and *cdkA* and *UBCE* displayed the lowest *M* values in OUT_GP, OUT_DC and IND samples, respectively. In ALL, the combination of the genes *EFL* and *RPS10* was the best. As a whole, the least stable genes were *eIF2-1* and *PGK*, although unexpectedly the former had the most stable expression in IND samples. To determine the optimal number of reference genes that would be required for an accurate normalization of gene expression analysis, pairwise variation values (*V*) were calculated with the cut-off of suitability set at 0.15, and below this value the addition of an additional internal reference gene does significantly improve normalization. As shown in Fig 3, the *V*2/3 was below the cut-off for all the experimental conditions studied and for the global analysis ALL. Thus, at least two genes were predicted as necessary for optimal normalization of gene expression levels in our experimental set-up.

#### NormFinder analysis

**Fig 4:**
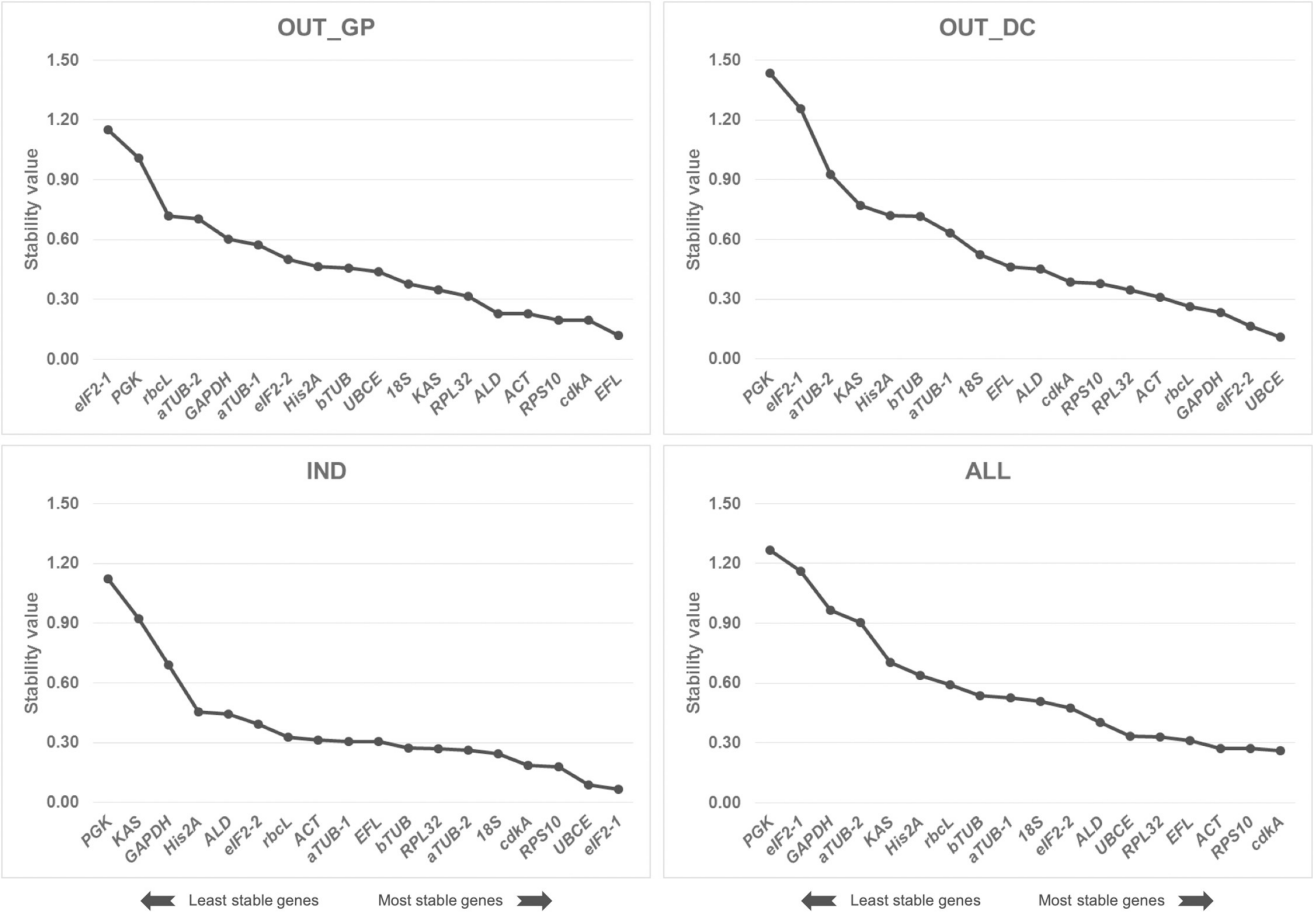
Global ranking of the 18 candidate reference genes using NormFinder analysis. The expression stability value was determined in all tested samples (a total of 45) and conditions (OUT_GP: Outdoor cultures during the growth phase, OUT_DC: Outdoor cultures during the semi-continuous phase, IND: Indoor cultures, ALL: Combination of the three previous conditions). A lower value indicates a more stable expression pattern.

The stability of the 18 candidate reference genes evaluated with NormFinder software (Fig 4) should prevent misinterpretations of results owing to artificial selection of co-regulated genes. The stability ranking of candidate reference genes was significantly different between conditions. The top three stable candidate genes were *EFL*, *cdkA*, and *RPS10* in OUT_GP, while *UBCE*, *eIF2-2*, and *GAPDH* were ranked top in OUT_DC. In IND, *eIF2-1* was the most stable gene followed by *UBCE* and *RPS10*, although for OUT_GP, OUT_DC and ALL (pool of samples from all conditions) it was the least stable gene together with *PGK*. Finally, *cdkA* was the most stable gene in ALL followed by *RPS10* and *ACT*.

#### BestKeeper analysis

The BestKeeper algorithm is a Microsoft Excel-based software and calculates the standard deviation (SD) and the coefficient of variation (CV) based on *C*_T_ values. Candidate reference genes with lower SD and CV have higher stability and are more suitable for normalization and genes with SD > 1 are considered unstable and unsuitable for normalization. The results of the BestKeeper analysis are shown in Table 2. The top-ranked candidate genes in OUT_GP exhibited significantly higher SD (~2-fold) than the top-ranked genes in OUT_DC and IND. Based on SD values, the most stable genes were *UBCE* in OUT_GP, *RPL32* in OUT_DC, and *EFL* in IND and ALL. Moreover, *EFL* was also ranked among the top three genes in OUT_GP and OUT_DC. *RPL32* was ranked second in OUT_GP and ALL but ninth in IND, and *UBCE* ranked seventh in OUT_DC and eighth in IND. Overall, with the BestKeeper algorithm *PGK*, *eIF2-1* and *KAS* were the least stable genes.

**Table 2:** Stability values of candidate reference genes as determined by BestKeeper in the different culture conditions.

OUT_GP			OUT_DC			IND			ALL		
Gene symbol	SD	CV	Gene symbol	SD	CV	Gene symbol	SD	CV	Gene symbol	SD	CV
*UBCE*	0.48	2.11	*RPL32*	0.20	1.10	*EFL*	0.22	1.35	*EFL*	0.37	2.30
*RPL32*	0.53	2.91	*EFL*	0.25	1.57	*aTUB-1*	0.24	1.24	*RPL32*	0.40	2.24
*EFL*	0.56	3.34	*RPS10*	0.28	1.53	*ALD*	0.25	1.21	*UBCE*	0.53	2.29
*His2A*	0.59	2.62	*rbcL*	0.32	2.59	*eIF2-2*	0.33	1.32	*cdkA*	0.63	2.50
*rbcL*	0.60	4.76	*eIF2-2*	0.47	1.91	*aTUB-2*	0.33	1.44	*RPS10*	0.63	3.36
*cdkA*	0.65	2.54	*GAPDH*	0.47	1.86	*cdkA*	0.33	1.33	*rbcL*	0.65	5.08
*aTUB-2*	0.75	3.07	*UBCE*	0.62	2.73	*GAPDH*	0.37	1.53	*eIF2-2*	0.72	2.86
*bTUB*	0.75	3.37	*18S*	0.68	6.44	*UBCE*	0.43	1.84	*ALD*	0.73	3.41
*aTUB-1*	0.78	3.91	*ACT*	0.78	3.60	*RPL32*	0.44	2.45	*aTUB-1*	0.77	3.97
*ACT*	0.81	3.57	*His2A*	0.81	3.63	*eIF2-1*	0.44	1.66	*18S*	0.77	6.87
*eIF2-2*	0.83	3.15	*cdkA*	0.90	3.64	*His2A*	0.63	2.70	*ACT*	0.78	3.50
*18S*	0.85	7.63	*aTUB-2*	0.93	3.72	*RPS10*	0.68	3.60	*His2A*	0.78	3.45
*RPS10*	0.85	4.48	*ALD*	0.93	4.40	*ACT*	0.69	3.10	*aTUB-2*	0.94	3.91
*ALD*	0.89	4.04	*KAS*	1.03	4.51	*18S*	0.70	6.00	*bTUB*	0.98	4.44
*KAS*	0.95	4.05	*aTUB-1*	1.07	5.73	*bTUB*	0.82	3.67	*KAS*	1.15	4.96
*GAPDH*	1.35	5.02	*bTUB*	1.18	5.51	*rbcL*	0.82	6.19	*GAPDH*	1.19	4.68
*eIF2-1*	1.44	4.93	*eIF2-1*	1.65	6.16	*KAS*	1.51	6.57	*eIF2-1*	1.42	5.17
*PGK*	1.62	7.51	*PGK*	1.72	8.31	*PGK*	1.72	7.70	*PGK*	1.80	8.36

OUT_GP: Outdoor cultures during the growth phase, OUT_DC: Outdoor cultures during the semi-continuous phase, IND: Indoor cultures, ALL: Combination of the three previous conditions. SD: Standard deviation, CV: Coefficient of variation.

#### Comparative Δ*C*_T_ analysis

**Fig 5:**
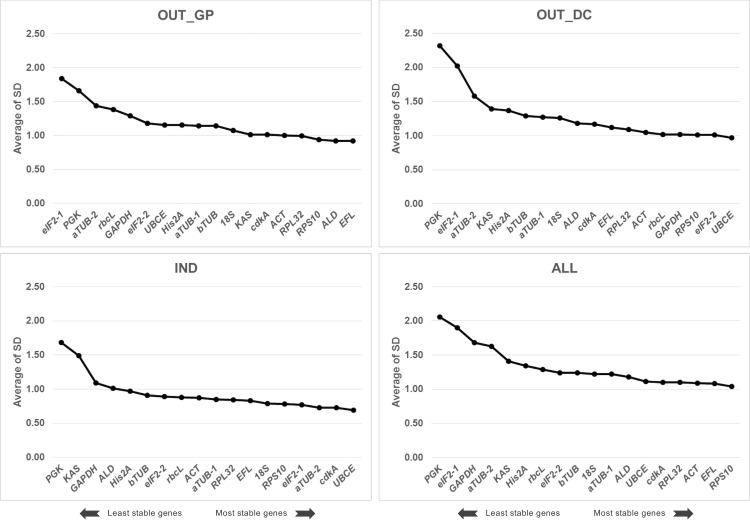
Global ranking of the 18 candidate reference genes using the comparative Δ*C*_T_ method. The expression stability value was determined in all tested samples (a total of 45) and conditions (OUT_GP: Outdoor cultures during the growth phase, OUT_DC: Outdoor cultures during the semi-continuous phase, IND: Indoor cultures, ALL: Combination of the three previous conditions). A lower value indicates a more stable expression pattern.

The comparative Δ*C*_T_ method uses the average standard deviation (SD) value of candidate reference genes as an indicator of expression stability. Genes with the lowest SD values are the most stable. As a whole, the highest stability of reference genes was found for IND samples and the 14 stable genes had an SD value < 1, compared to four reference genes for OUT_GP samples and one reference gene for OUT_DC samples (Fig 5). *EFL* ranked first in OUT_GP samples and *UBCE* was the most stable gene in OUT_DC and IND samples. *RPS10* had the lowest SD in the pooled samples, ALL, and was always among the top five most stable genes across all experimental conditions OUT_GP, OUT_DC and IND.

#### Comprehensive ranking of RefFinder

**Fig 6:**
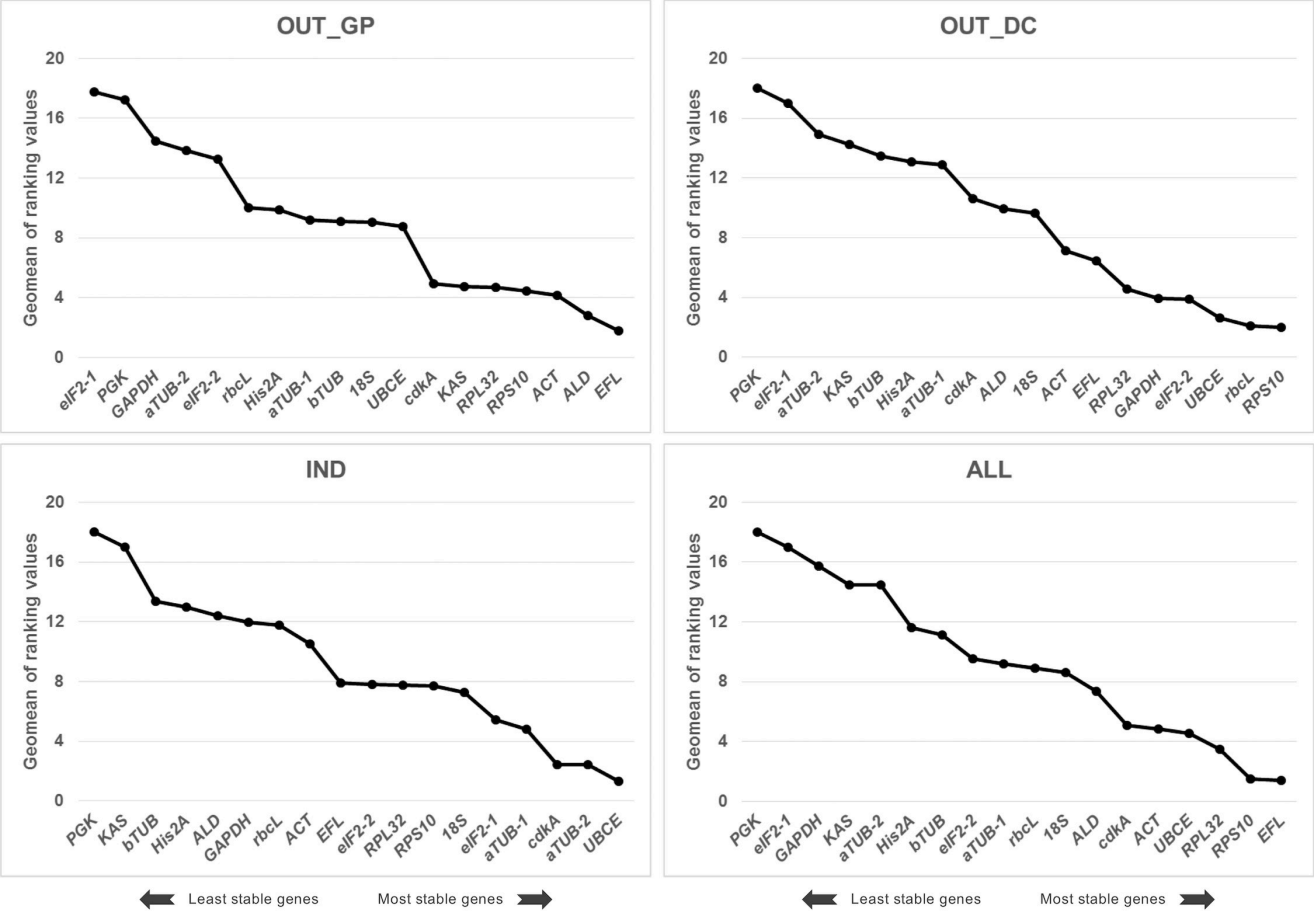
Comprehensive ranking of the 18 candidate reference genes using RefFinder. The expression stability value was determined in all tested samples (a total of 45) and conditions (OUT_GP: Outdoor cultures during the growth phase, OUT_DC: Outdoor cultures during the semi-continuous phase, IND: Indoor cultures, ALL: Combination of the three previous conditions). A lower value indicates a more stable expression pattern.

The web-based program RefFinder integrates the results of the four above-mentioned approaches (geNorm, NormFinder, BestKeeper and Δ*C*_T_ method), and calculates the geometric mean of the ranking of all the candidate reference genes included in the analysis. In OUT_GP samples, the most stable genes were *EFL*, *ALD* and *ACT*, whereas *RPS10*, *rbcL* and *UBCE* were the most suitable reference genes in OUT_DC (Fig 6). In IND, *UBCE*, *aTUB-2* and *cdkA* exhibited the highest expression stability. And in pooled samples, ALL, *EFL*, *RPS10* and *RPL32* were the most stable genes.

In summary, quite different rankings of the best candidate reference genes were observed when experimental samples from different culture conditions were compared and analyzed using different methods, which highlights that the results can vary significantly with the algorithm used. The geNorm output indicated that two reference genes were enough for normalization in all the conditions tested. Comparison of the ranking of the candidate reference genes with the different methods, revealed that the optimum references genes were *ALD* and *EFL* for OUT_GP, *RPL32* and *UBCE* for OUT_DC and *cdkA* and *UBCE* for IND. Moreover, *EFL* and *cdkA* or *EFL* and *UBCE* were the most appropriate for the pooled samples (ALL). In all the above recommendations, pairs of genes belonging to the same functional group were excluded to avoid possible existence of co-regulation that might interfere with the reliability of the results. For instance, although *EFL*, *RPS10* and *RPL32* were among the most stable genes for ALL, these three genes are involved in protein biosynthesis and hence only *EFL* (as top-ranked by geNorm, BestKeeper and RefFinder) was chosen.

### Expression profile of *AGPL* and *AGPS* in OUT_DC

**Fig 7:**
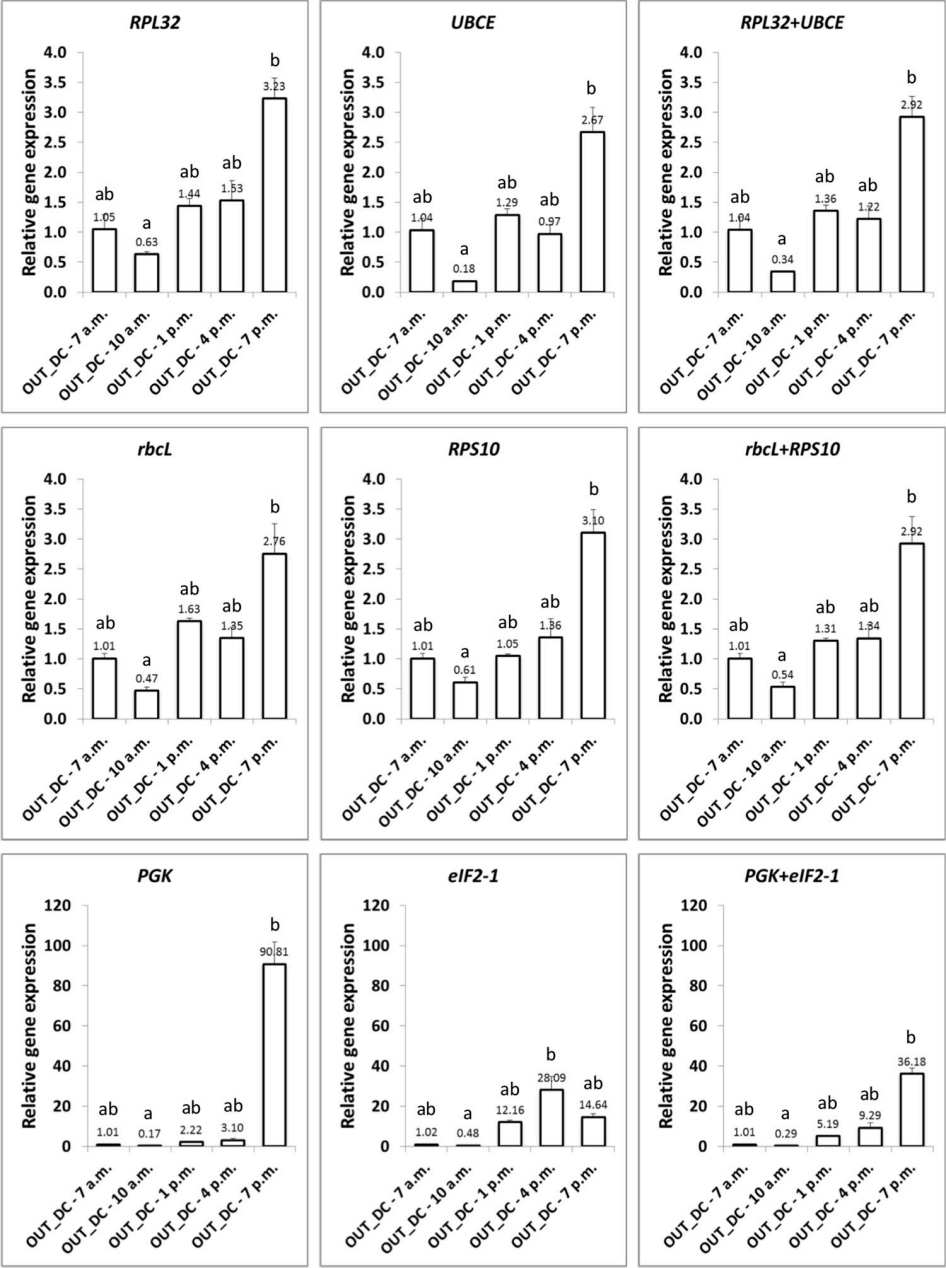
Relative *AGPL* expression profiles in OUT_DC samples determined by qRT-PCR using different reference genes. In all instances, data are expressed (at the outside end of the corresponding column) as the mean fold change (mean + SEM, n = 3) from the calibrator group (OUT_DC—7 a.m.). Different letters denote significant differences (*P* < 0.05) between time points using the Friedman test (non-parametric one-way ANOVA) followed by the Dunn’s multiple comparison test.

ADP-glucose pyrophosphorylase catalyzes the first step of starch biosynthesis, that is, the synthesis of ADP-glucose. In eukaryotes, it is a heterotetramer comprised of two distinct subunits, two alpha (AGPS) and two beta (AGPL). As starch accumulates due to carbon fixation during photosynthesis and is further degraded in the dark, we hypothesized that *AGPS* and *AGPL* were likely co-regulated and would have similar expression patterns modulated by starch content fluctuations. To test this hypothesis and verify the validated reference genes in RT-qPCR, we quantified *AGPL* and *AGPS* expression in OUT_DC samples. The reference genes for normalization were *RPL32* and *UBCE*, since they were the most stable for the OUT_DC samples and this was compared with the outcome of normalization with the least stable genes *PGK* and *eIF2-1*. In addition, *AGPL* and *AGPS* expression was normalized with *RPS10* and *rbcL*, the most stable reference genes for OUT_DC samples using the RefFinder program ranking. As a complementary analysis and considering the results obtained in the combined condition ALL, the suitability of other candidate reference genes such as *cdkA* and *ACT* in combination with *EFL* was also evaluated. As shown in Fig 7, when *RPL32* and *UBCE* genes were used for normalization, either alone or in combination, a statistically significant increase in *AGPL* transcripts was observed at 7 p.m. being 2.67-fold (*UBCE*) and 3.23-fold (*RPL32*) higher than at 7 a.m. (the calibrator time point). The gene expression results obtained with *rbcL*, *RPS10* and their combination matched those previously mentioned, with a peak in transcript abundance at 7 p.m. (ranging between 2.76-fold and 3.10-fold higher than at 7:00 a.m. with *rbcL* and *RPS10*, respectively). This profile was also conserved when the data were analyzed with *EFL* and *cdkA*, or *EFL* and *ACT* (S6 Fig), with some slight differences in the ratios. However, when the analysis was performed with the least stable genes *PGK* and *eIF2-1*, strong differences could be found. Although the expression pattern with *PGK* was similar to those found with stable genes in the sense that a peak in gene expression was detected at 7 p.m. and the lowest transcript levels were found at 10 a.m., the ratios were significantly different. Up to 90.81-fold higher transcripts were measured at 7 p.m., representing a higher than 500 fold-increase in relation to 10 a.m., this ratio being significantly higher than the ratios found with the most stable genes (always lower than 25-fold). Moreover, when *eIF2-2* was employed as internal control, it was striking that the highest expression levels of *AGPL* were detected at 4 p.m. (~28-fold higher than at 7 a.m.), although the lowest transcript amounts were measured again at 10 a.m. (0.48-fold lower than at 7 a.m.). With the combination of *PGK* and *eIF2-1* the maximum and minimum expression levels were observed again at 7 p.m. and 10 a.m. respectively, but with ratios not only at 7 p.m. but also at 1 p.m. and 4 p.m. being strongly different to those shown with the most stable genes. As a whole, analyses with stable reference genes fitted our hypothesis in the sense that the highest transcript amounts of *AGPL* in OUT_DC were observed at the end of the light phase, when maximum starch accumulation might be expected.

**Fig 8:**
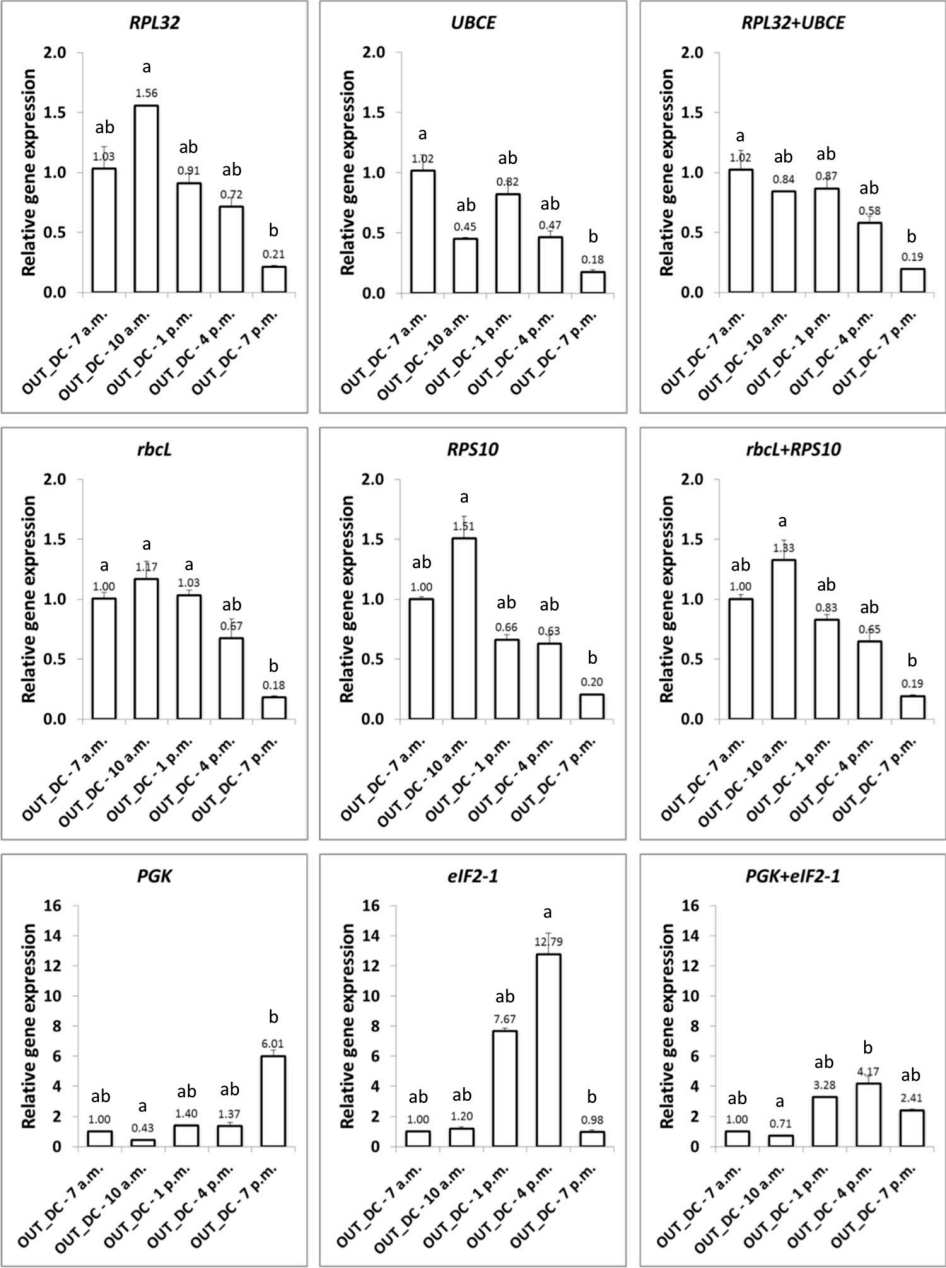
Relative *AGPS* expression profiles in OUT_DC samples determined by qRT-PCR using different reference genes. In all instances, data are expressed (at the outside end of the corresponding column) as the mean fold change (mean + SEM, n = 3) from the calibrator group (OUT_DC—7 a.m.). Different letters denote significant differences (*P* < 0.05) between times points using the Friedman test (non-parametric one-way ANOVA) followed by the Dunn’s multiple comparison test.

The expression of *AGPS* was evaluated using the same reference genes for normalization as was used for *AGPL* (Fig 8). The *AGPS* gene expression profile and expression ratios after normalization with *RPL32* and *UBCE* alone or in combination, revealed a significant down-regulation at 7 p.m. (~5-fold lower transcripts) compared to 7 a.m. (used as a calibrator). Similar results were also obtained for *AGPS* expression normalized with *rbcL*, *RPS10* and their combination. The large drop in *AGPS* transcription at 7:00 p.m. was even more pronounced (~6-fold lower than at 7:00 a.m.) when *EFL* and *cdkA* or *EFL* and *ACT* were used for normalization (S6 Fig). However, expression patterns were substantially modified when normalization was performed using the least stable reference genes. For example, a significant up-regulation of *AGPS* was observed at 7 p.m. when *PGK* was used for normalizarion (6.01-fold higher than at 7 a.m.), and at 4 p.m. when *eIF2-1* was used for normalization (12.79-fold higher) or when both genes was used (4.17-fold higher). In contrast to what was initially hypothesized, transcriptional regulation of *AGPS* was opposite to that of *AGPL*, as the lowest transcript abundance of *AGPS* occurred concomitantly with the highest transcription of *AGPL* at the end of the light phase.

In conclusion, when appropriate reference genes are selected for normalization in gene expression analysis using different algorithms similar outcomes are obtained. However, inappropriate selection of reference genes is a major concern as it can lead to substantially modified gene expression patterns.

## Discussion

In this stydy, the expression stability of 18 candidate reference genes was evaluated in the green microalgae species *T*. *chui* using geNorm, NormFinder, BestKeeper, the comparative Δ*C*_T_ method, and RefFinder. Three different experimental conditions for microalgae production were tested (OUT_GP, OUT_DC, and IND), as well as a pool of all samples (ALL). The most prominent observations was that each of the above tools produced a different set of top-ranked reference genes in each group of experimental samples and conditions. This finding was not entirely unexpected, as such heterogenous results may be caused by application of different mathematical algorithms as found in previous studies [21,33,35,37]. As each algorithm has its own drawbacks [63], the use of different methods to define the most appropriate reference genes for target gene expression normalization in RT-qPCR analysis is strongly recommended to improve the reliability of results. Moreover, to our knowledge, the present study represents the first RT-qPCR survey of candidate genes for normalization *in T*. *chui*. The only related study was focused on the expression of a high-affinity phosphate transporter gene in *T*. *chui* using absolute rather than relative PCR quantification so that no normalization was needed [64]. Absolute quantification requires construction of a standard sense RNA for every target gene of interest to create an external calibration curve, and this other well-known constraints of absolute quantification [65], makes it significantly less practical when quantifying ten or more genes in a single study. Herein the suitability of 18 candidate reference genes for relative real-time expression analysis in microalgae samples harvested in a production setting from 4000 L photobioreactors under different conditions, OUT_GP and OUT_DC, was evaluated. The main stress conditions for microalgae were related to the daily environmental changes, with light intensity and temperature being probably the most relevant factors. This study aimed to have a final application in industry and as such is very different from the highly controlled, low culture volumes of laboratory conditions, either standard or stressful, previously used to identify reference for other microalgae species [44,47,49–53,66].

Gene expression analyses represent a powerful tool for gaining insight into the molecular and biochemical mechanisms that govern physiological features and responses to environmental changes in a wide range of different organisms, including microalgae. For this reason high throughput sequencing approaches have been employed to provide a comprehensive view of the responsiveness of different metabolic pathways under defined experimental conditions, nitrogen starvation being probably the most widely assessed [58,67–69]. Once the genetic resources containing thousands of predicted transcripts are generated and become available, such information can be further processed and used to study the expression of selected sets of genes involved in relevant metabolic pathways through RT-qPCR platforms containing a variable number of representative genes. For instance, 16 differentially expressed genes involved in lipid accumulation and carbon fixation were studied in the species *Chorella sorokiniana* grown under nitrogen limiting conditions to validate RNA-seq data [70]. In *Tetraselmis suecica*, a set of 14 selected genes exhibited significant up- and down-regulation using RT-qPCR in agreement with RNA-seq results [58]. Recently, a RT-qPCR platform containing more than 100 targeted genes was used for *Nannochloropsis gaditana* to study the influence of light quality on metabolism [71]. A similar strategy with an array of ~100 genes was also employed in human cell lines to screen for the potential bioactivity of microalgae extracts [72]. These studies demonstrate that modulation of metabolic processes can be detected via gene expression analysis of gene markers. When dealing with microalgae production at an industrial scale, expression analysis with a species-specific RT-qPCR platform containing selected gene markers represents and innovative biotechnological approach for quality control of biomass. For *T*. *chui*, although there are available genetic resources, biotechnological tools still need to be developed. The present study identifying appropriate reference genes represents a first and key step for the accurate detection of varying gene expression under different conditons.

Most of the candidate reference genes included in this study have previously been tested in other microalgae species. In particular, the stability of the traditional *GAPDH*, *ACT*, and *18S* reference genes has been simultaneously assessed in a range of microalgae species [17,43,47,49,50,52,53,55,70], macroalgae [40,73,74] and plants [22,75,76]. The two candidate alpha tubulin encoding genes here analyzed, *aTUB-1* and *aTUB-2*, probably arose by gene duplication and this observation is not restricted to *T*. *chui*. Tubulin belongs to a multigene family in other eukaryotes including mammals, ciliates, fungi, or plants [77–80], and a variable number of gene duplicates have also been found in other evolutionary distant microalgae such as *Bigelowiella*, *Cyanophora*, *Emiliania* or *Chlamydomonas* [81]. Two genes encoding the eukaryotic translation initiation factor 2 (*eIF2-1* and *eIF2-2*) were identified in *T*. *chui* and were analyzed. The eIF2-2 isoform in *T*. *chui* was most similar to mitochondrial isoforms, but lacked a signal peptide as shown by analysis with SignalP 5.0 [82]. To our knowledge, this is the first time that these gene duplicates have been tested as reference genes in microalgae, as the eukaryotic translation initiation factor 4E (*eIF4E*) was assessed in the dinoflagellates *Prorocentrum minimum* [47] and *Karenia mikimotoi* [50], and another gene referred to as eukaryotic (translation) initiation factor was tested in the green microalgae *Closterium ehrembergii* [56] or the dinoflagellate *Alexandrium catanella* [49].

The *EFL* gene included in this study is related to, but clearly distinguishable from the eukaryotic elongation factor-1 alpha gene (*EF-1α*) previously analyzed in several microalgae [52–54]. *EF-1α* expression was also assessed in green microalgae such as *Volvox carteri* [55] and *Chlamydomonas* [17,57], although since only *EFL* has been reported in green algae it seems likely that the putative *EF-1α* was misannotated [66,83]. In addition, the inclusion in the present study of *KAS* as a potential reference gene for normalization is new to microalgae, and came from previous data about gene stability in the microalgae *Nannochloropsis gaditana* [84]. Finally, the remaining genes (*bTUB*, *PGK*, *His2A*, *rbcL*, *cdkA*, *UBCE*, *ALD*, *RPL32* and *RPS10*) were selected as they have been previously tested as reference genes in microalgae.

The results for the candidate reference genes in *T*. *chui* comparted to previous studies of other microalgae species revealed they were variable. For instance, although *GAPDH* and *ACT* are the most used reference genes for normalization in metazoan [26], few studies have demonstrated them to be optimal for normalization. For example, *GAPDH* was the most stable gene under different experimental conditions in *Chlamydomonas* [17,57], but in the present study it was not top-ranked. In contrast, *ACT*, which led the stability ranking in *Chlamydomonas* sp. [17], *Nannochloropsis* sp. [52], *Ditylum brightwellii* [45], *Closterium ehrenbergiii* [56], *Isochrysis zhangjiangensis* [51], *Tetraselmis suecica* [58], and was the second most stable gene in *Alexandrium catanella* [49], was not well ranked in our study or *Volvox* [55], Similarly, *18S* was not the most stable gene in any condition in *T*. *chui*.

The use of alpha and beta tubulin genes has been has been reported in other microalgae species and in some cases these genes were two of the most stable genes [44]. In other studies only one of the two genes was stable [47,50–52,54,56,58], and in other microalgae species none of the two genes was among those with the highest stability [17,43,55,57]. In this study, *bTUB* was never among the top seven most stable genes in any of the analysis or conditions assayed, and with the exception of IND, neither *aTUB-1* nor *aTUB-2* was in the first seven ranked genes. Overall, our study revealed that choice of the reference gene depends on species, experimental conditions and the analytical approach, highlighting the need for appropriate stability testing of reference genes before their use in expression analysis of target genes.

From an industrial perspective we assessed the expression of the *T*. *chui* genes encoding both the large (*AGPL*) and small (*AGPS*) subunits of ADP-glucose pyrophosphorylase, as the functional enzyme is a hetero-tetrameric protein composed of two alpha (or small, S) and two beta (or large, L) subunits [85,86]. The results confirmed that the use of inappropriate reference genes can lead to strong differences not only in the expression profile of the target gene, but also in its relative expression levels (expressed as fold-changes) between sampling points. This is in agreement with the findings of previous studies [17,52,54,87], which highlights the importance of validating reference gene stability. The second main objective was to study possible transcriptional regulation of both genes in an outdoor production system. The ADP-glucose pyrophosphorylase is involved in the synthesis of ADP-glucose, which is the donor for the elongation of alpha-1,4-glucosidic chains that serve as intracellular carbon and energy storage, as starch in plants and green and red algae and glaucophytes [88,89]. In microalgae, starch production can be strongly affected by light intensity and temperature [90,91], factors that significantly varied in samples collected from OUT_DC. Although *AGPL* and *AGPS* are both involved in starch synthesis the genes had a different regulation, as the peak of *AGPL* expression by the end of the light phase coincided with the lowest expression levels of *AGPS*. In a diurnal cycle, an increase in transcripts of ADP-glucose pyrophosphorylase gene was reported for the picoalgae *Ostreococcus* [92]. This finding suggests that *AGPL* and *AGPS* have different daily rhythms in *T*. *chui* under natural photoperiod. In this regard, genes exhibiting differential transcriptional regulation under light/dark cycles have been reported in a range of different microalgae such as *Chlamydomonas* [93,94], *Ostreococcus* [93,95], *Nannochloropsis* [96], *Phaeodactylum* [97], *Tetradesmus* [98], *Bigelowiella* [99], or *Chrysochromulina* [100]. More research will be needed to confirm if the transcriptional pattern of *AGPL* and *AGPS* is conserved in a diel cycle, as well as to analyze the effect of changing climate conditions on their expression.

## Conclusions

As far as we are aware the present study represents the first systematic analysis of candidate reference genes for the species *T*. *chui*. Moreover, and in contrast to all previous reports on microalgae, the samples analyzed were collected from large scale outdoor photobioreactors from operating industrial production units. For each of the conditions assessed (OUT_GP, OUT_DC, and IND), distinct rankings of genes were obtained with the tested algorithms. Overall, the following references genes were identified as stably-expressed: *ALD* and *EFL* for OUT_GP, *RPL32* and *UBCE* for OUT_DC, and *cdkA* and *UBCE* for IND. Moreover, the genes *EFL* and *cdkA* or *EFL* and *UBCE* were the most appropriate for pooled samples (ALL). Expression analysis of the genes *AGPL* and *AGPS* in OUT_DC samples confirmed that the selected reference genes were appropriate for normalization of target genes and the consequence of inappropriate reference genes for accuracy and reliability of the quantification. Finally, the present study forms the basis of an RT-qPCR platform to be used for quality control of *T*. *chui* biomass in industrial production facilities.

## Supporting information

S1 Fig Evolution of cell density in indoor cultures. The average ± SD of three 5 L flasks is represented. Samples were collected after inoculation on day 1 (D1), and then at D4, D7, D10 and D13.
(TIF)

S2 Fig Evolution of cell density in outdoor cultures. The average ± SD of three 4000 L photobioreactors of the same production unit is represented. Samples were collected after inoculation on day 1 (D1), and then at D4, D7, D11 and D18.
(TIF)

S3 Fig Agarose gel electrophoresis of PCR amplified products. The expected size of each amplicon (in bp) is shown. The 25 bp DNA Ladder (Invitrogen) was used as the molecular weight standard and the size (in bp) of the most relevant bands are shown. The black dashed line in the gel of the lower panel (TARGET GENES) indicates where irrelevant lanes were removed.
(TIF)

S4 Fig Standard curves generated by all primer pairs. The curves were used for the determination of PCR efficiencies. Serial dilutions of input cDNA were plotted against the *C*_T_ values obtained by real-time PCR.
(TIF)

S5 Fig Melting curves generated by all primer pairs used in the study. The melt curves were captured after cycle 40 by heating from 70°C to 95°C with a ramp speed of 0.5 s every 10 s.
(TIF)

S6 Fig Relative *AGPL* (upper panel) and *AGPS* (lower panel) expression profiles in OUT_DC samples. The expression of *AGPL* and *AGPS* were determined by RT-qPCR using different reference genes. In all instances, data are expressed (at the outside end of the corresponding column) as the mean fold change (mean + SEM, n = 3) from the calibrator group (OUT_DC—7 a.m.). Different letters denote significant differences (*P* < 0.05) between time points using the Friedman test (non-parametric one-way ANOVA) followed by the Dunn’s multiple comparison test.
(TIF)

S1 Table Culture temperature (average ± SD,°C) and PAR (μmol m^-2^ s^-1^) figures at sampling days and times in outdoor conditions. (DOCX)

S2 Table Candidate reference genes for normalization of RT-qPCR expression data. Information regarding the target genes *AGPL* and *AGPS* included in the study is also shown below.
(DOCX)

S3 Table Mean *C*_T_ values of all samples included in this study. Data are presented for each of the candidate reference genes.
(XLSX)

S1 Raw images (PDF)
