# Supplementary figures and images for: Selection and validation of reference genes for quantitative real-time PCR in the green microalgae Tetraselmis chui
Source: PLoS One. 2021 Jan 14;16(1):e0245495. doi: 10.1371/journal.pone.0245495 (PMC7808622; doi:10.1371/journal.pone.0245495)

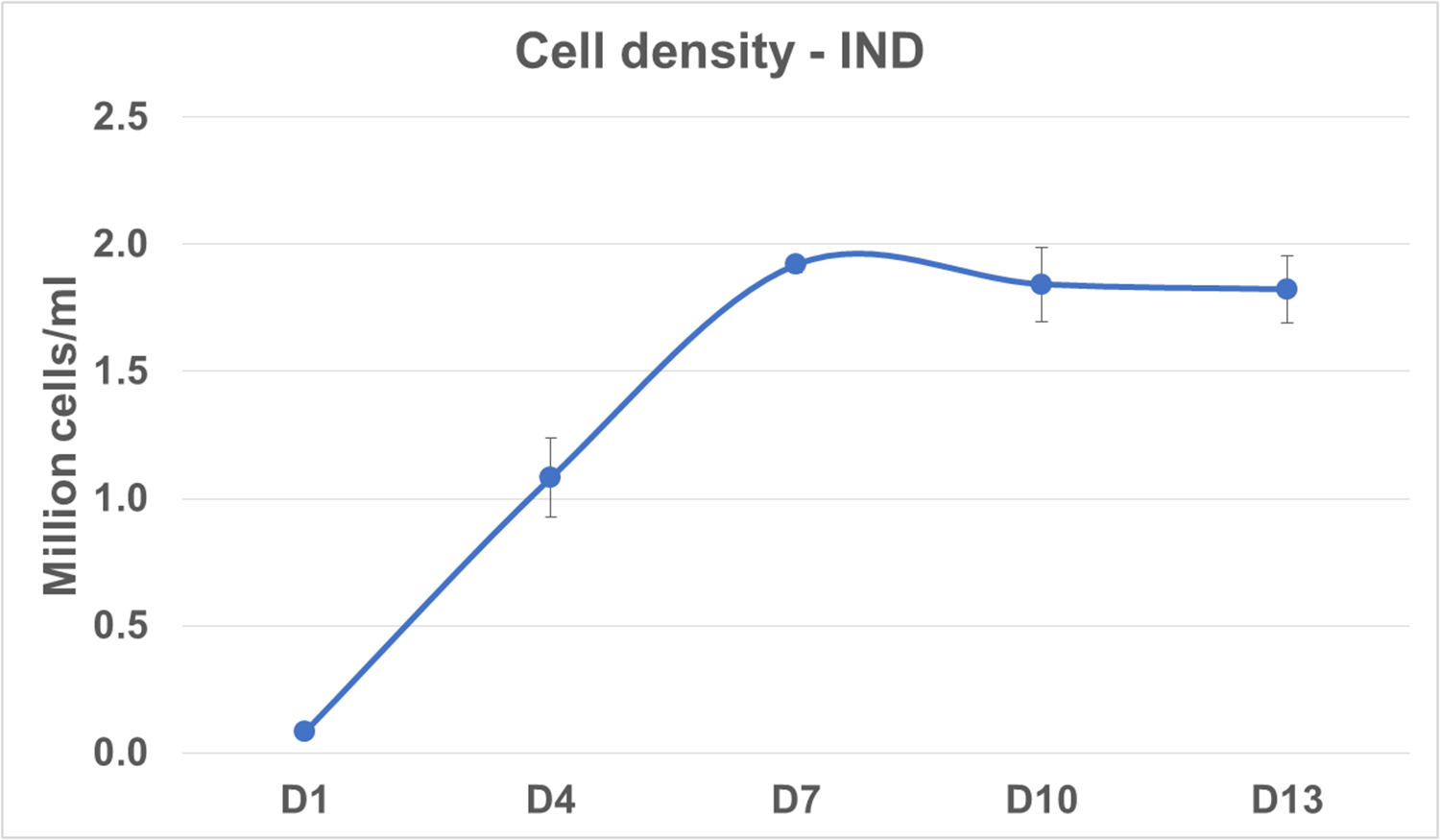

Supplement: S1 Fig — The average ± SD of three 5 L flasks is represented. Samples were collected after inoculation on day 1 (D1), and then at D4, D7, D10 and D13. (TIF) [file pone.0245495.s001.tif]

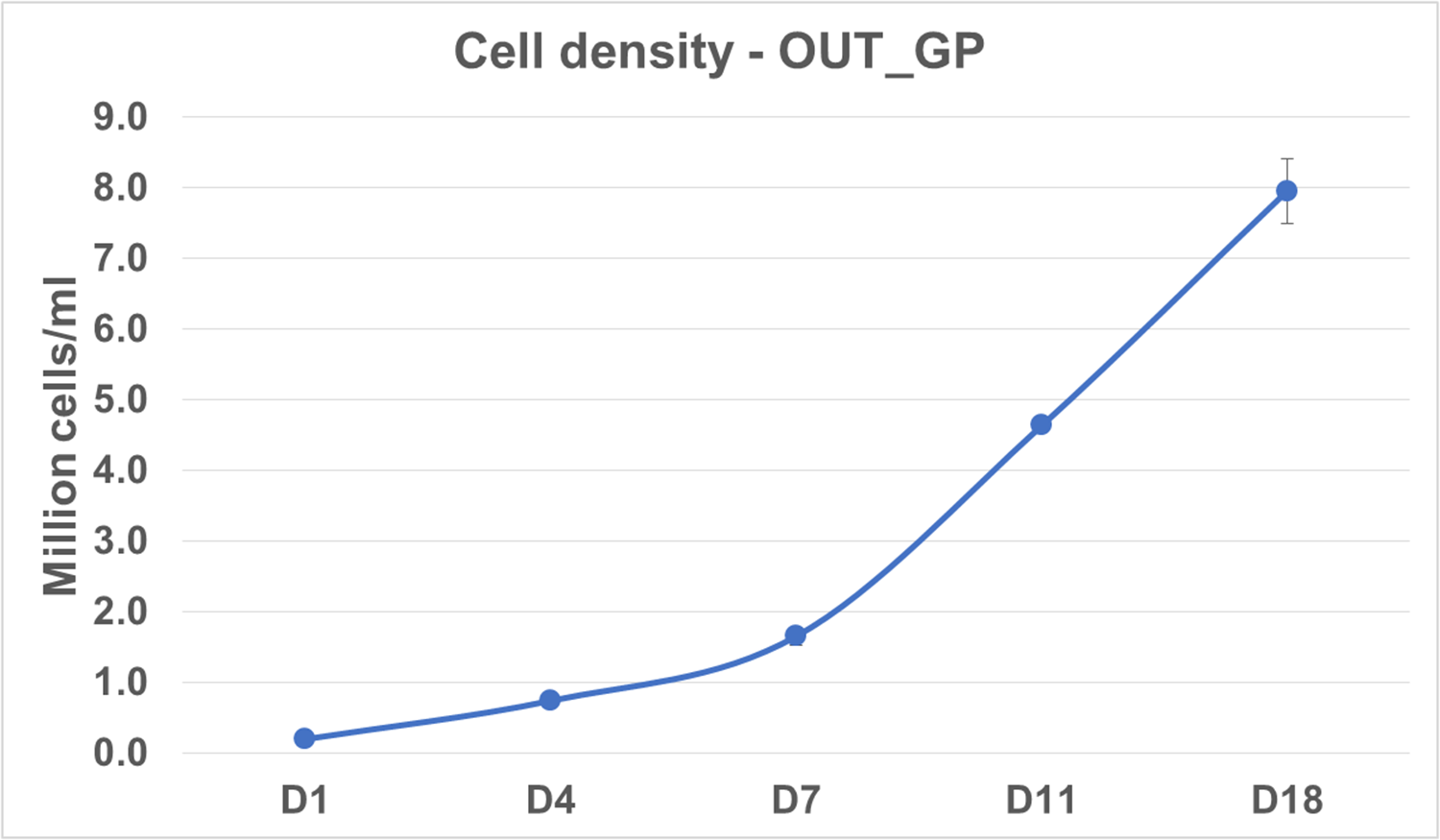

Supplement: S2 Fig — The average ± SD of three 4000 L photobioreactors of the same production unit is represented. Samples were collected after inoculation on day 1 (D1), and then at D4, D7, D11 and D18. (TIF) [file pone.0245495.s002.tif]

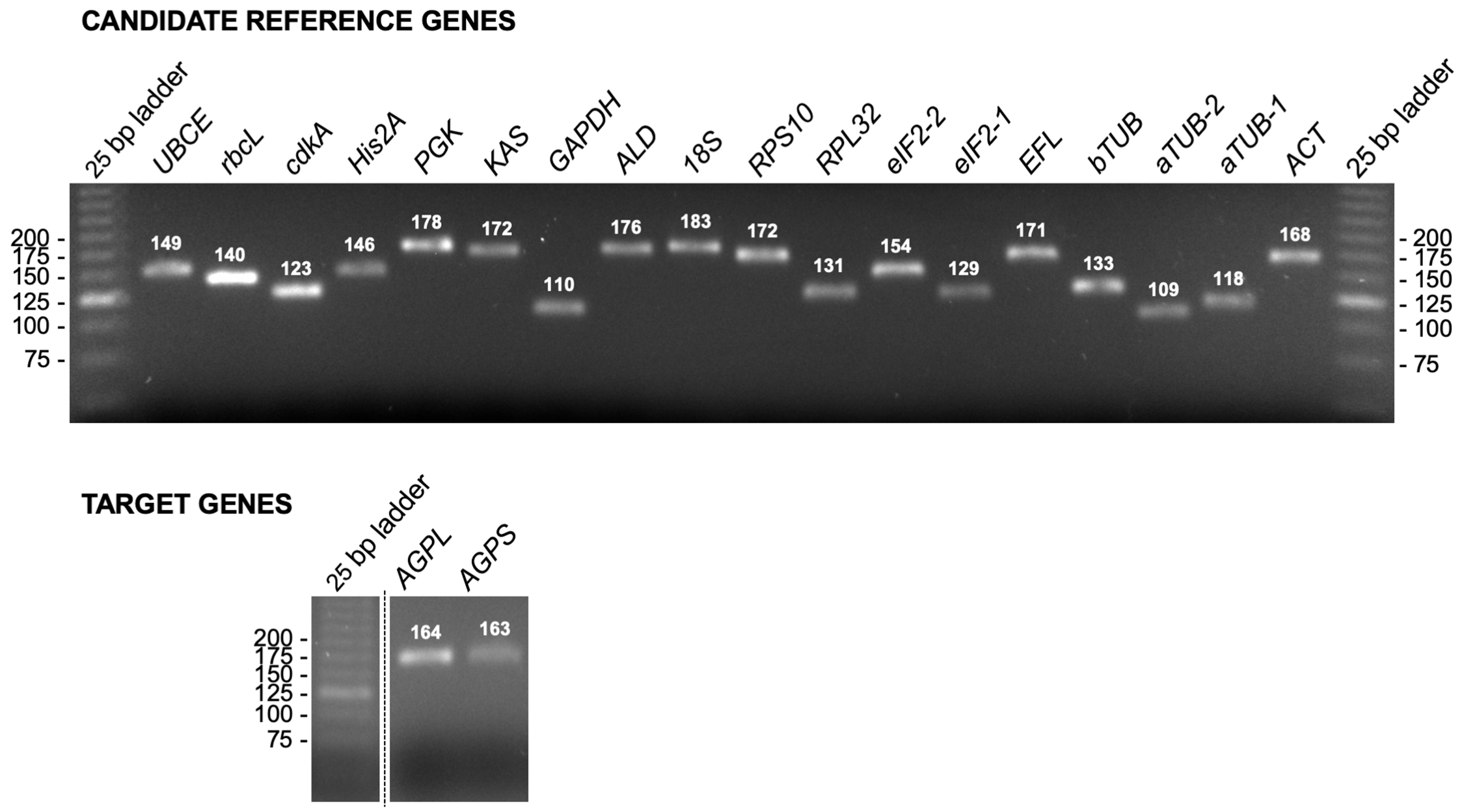

Supplement: S3 Fig — The expected size of each amplicon (in bp) is shown. The 25 bp DNA Ladder (Invitrogen) was used as the molecular weight standard and the size (in bp) of the most relevant bands are shown. The black dashed line in the gel of the lower panel (TARGET GENES) indicates where irrelevant lanes were removed. (TIF) [file pone.0245495.s003.tif]

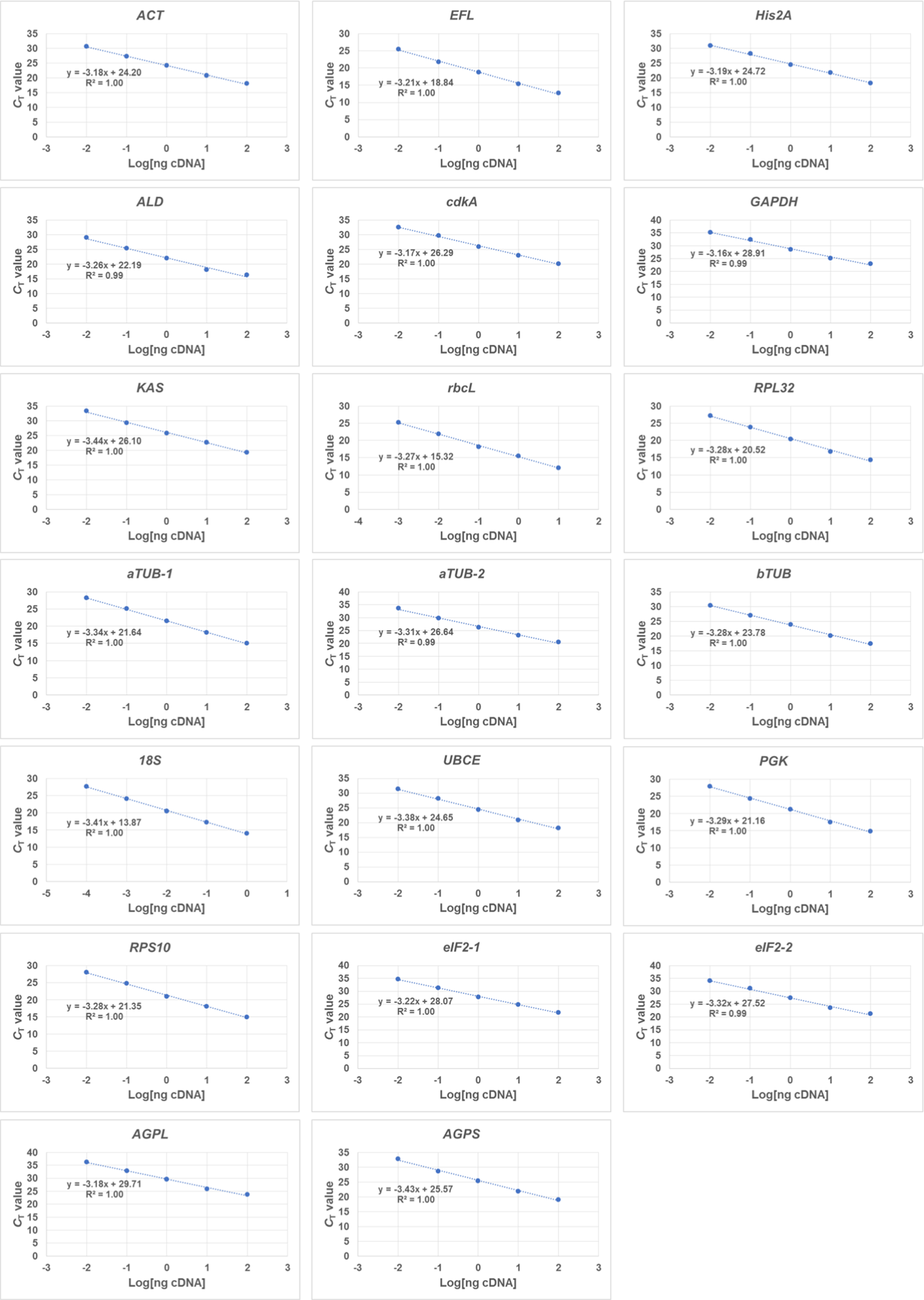

Supplement: S4 Fig — The curves were used for the determination of PCR efficiencies. Serial dilutions of input cDNA were plotted against the CT values obtained by real-time PCR. (TIF) [file pone.0245495.s004.tif]

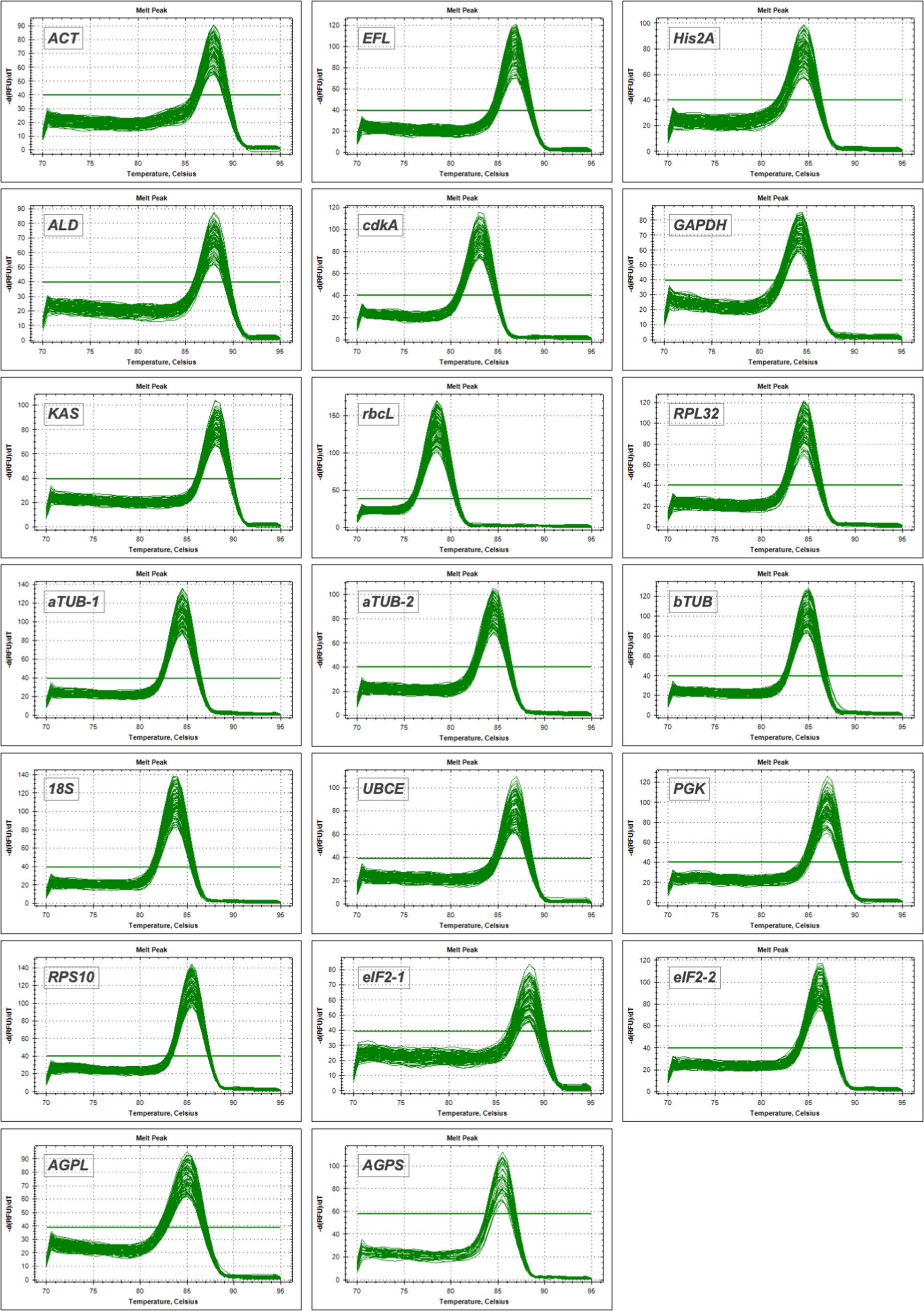

Supplement: S5 Fig — The melt curves were captured after cycle 40 by heating from 70°C to 95°C with a ramp speed of 0.5 s every 10 s. (TIF) [file pone.0245495.s005.tif]

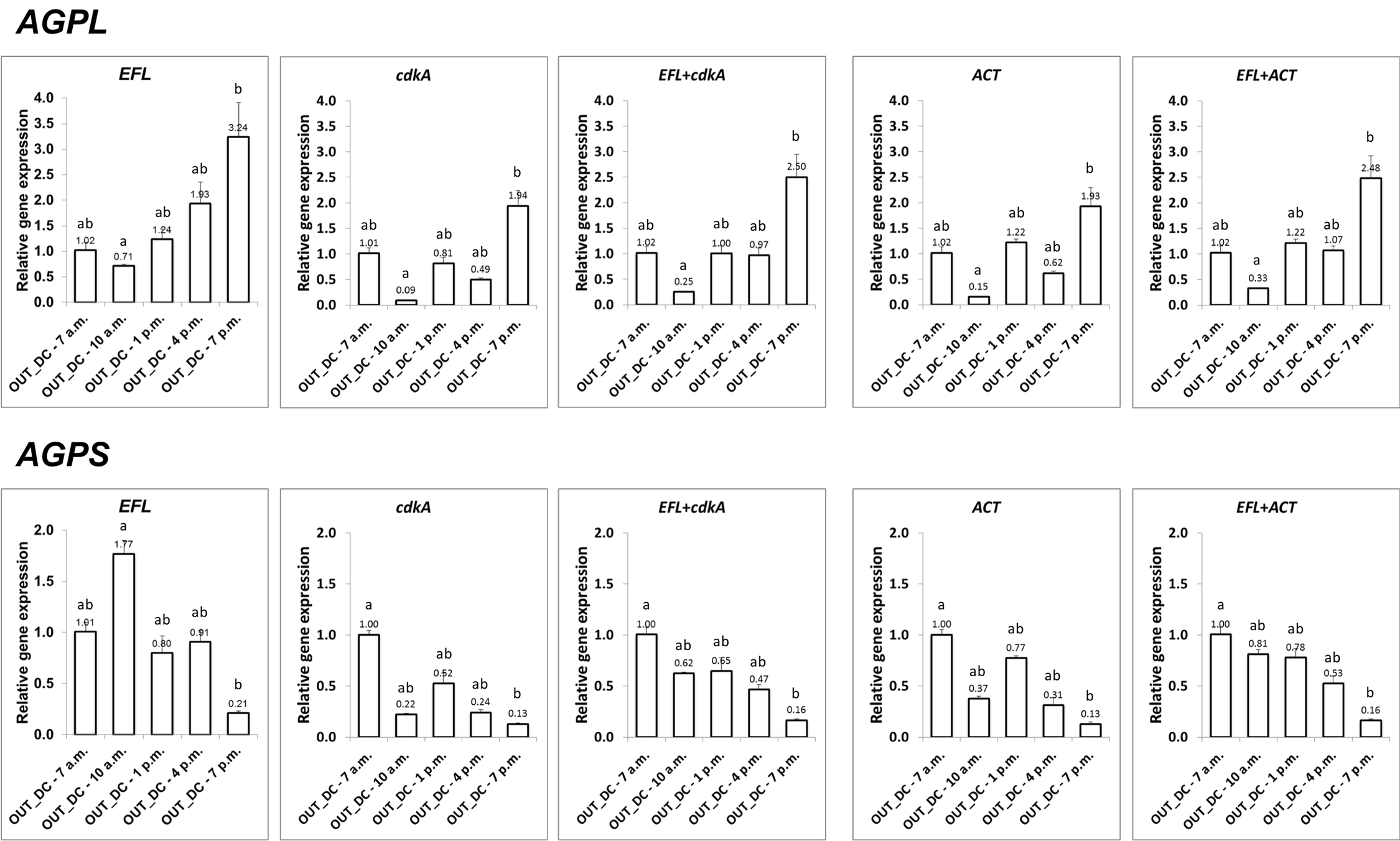

Supplement: S6 Fig — The expression of AGPL and AGPS were determined by RT-qPCR using different reference genes. In all instances, data are expressed (at the outside end of the corresponding column) as the mean fold change (mean + SEM, n = 3) from the calibrator group (OUT_DC—7 a.m.). Different letters denote significant differences (P < 0.05) between time points using the Friedman test (non-parametric one-way ANOVA) followed by the Dunn’s multiple comparison test. (TIF) [file pone.0245495.s006.tif]
