# Supplementary material for: Selection and validation of reference genes for quantitative real-time PCR in the green microalgae Tetraselmis chui
Source: PLoS One. 2021 Jan 14;16(1):e0245495. doi: 10.1371/journal.pone.0245495 (PMC7808622; doi:10.1371/journal.pone.0245495)
Supplement: S1 Table — (DOCX) [file pone.0245495.s007.docx]

| **OUT_GP** |  |  |  |
| --- | --- | --- | --- |
| Day | Time | Temperature | PAR |
| D1 | 1:00 p.m. | 15.3 ± 1.4 | 810 |
| D4 | 1:00 p.m. | 14.4 ± 1.2 | 450 |
| D7 | 1:00 p.m. | 20.3 ± 1.3 | 1400 |
| D11 | 1:00 p.m. | 17.8 ± 1.2 | 1100 |
| D18 | 1:00 p.m. | 18.7 ± 1.4 | 300 |
| **OUT_DC** |  |  |  |
| Time | Temperature | PAR |  |
| 7:00 a.m. | 10.6 ± 0.1 | 6 |  |
| 10:00 a.m. | 13.2 ± 0.3 | 970 |  |
| 1:00 p.m. | 19.0 ± 1.3 | 1300 |  |
| 4:00 p.m. | 22.2 ± 0.5 | 580 |  |
| 7:00 p.m. | 19.8 ± 0.2 | 3 |  |
