# Supplementary material for: Selection and validation of reference genes for quantitative real-time PCR in the green microalgae Tetraselmis chui
Source: PLoS One. 2021 Jan 14;16(1):e0245495. doi: 10.1371/journal.pone.0245495 (PMC7808622; doi:10.1371/journal.pone.0245495)
Supplement: S2 Table — Information regarding the target genes AGPL and AGPS included in the study is also shown below. (DOCX) [file pone.0245495.s008.docx]

| CANDIDATE REFERENCE GENES | | | | |  |  |  |  |
| --- | --- | --- | --- | --- | --- | --- | --- | --- |
| Gene symbol | Sequence ID (*T. chui* PLY429) | Most relevant hit (BLASTp) | Species | Accession No | | Identity (%) | | |
| *ACT* | MMETSP0491_2-20121128\|5070_1 | Actin | *C. reinhardtii* | XP_001699068 | | 89 | | |
| *EFL* | MMETSP0491_2-20121128\|385_1 | Elongation factor-1 alpha-like | *T. tetrathele* | ABQ81944 | | 94 | | |
| *His2A* | MMETSP0491_2-20121128\|6926_1 | Histone 2A variant | *C. reinhardtii* | XP_001693700 | | 91 | | |
| *ALD* | MMETSP0491_2-20121128\|16018_1 | Fructose-1,6-biphosphate aldolase, class II | *O. tauri* | OUS43358 | | 63 | | |
| *cdkA* | MMETSP0491_2-20121128\|4820_1 | Cell division control protein 2 homolog A isoform X1 | *M. acuminata subsp. malaccensis* | XP_009412825 | | 73 | | |
| *GAPDH* | MMETSP0491_2-20121128\|510_1 | Glyceraldehyde-3-phosphate dehydrogenase | *C. reinhardtii* | XP_005848099 | | 66 | | |
| *KAS* | MMETSP0491_2-20121128\|9439_1 | Beta-ketoacyl acyl carrier protein synthase | *H. lacustris* | AEF13159 | | 68 | | |
| *RPL32* | MMETSP0491_2-20121128\|11_1 | 60S ribosomal protein L32-1 | *A. protothecoides* | XP_011396614 | | 74 | | |
| *aTUB-1* | MMETSP0491_2-20121128\|9056_1 | Tubulin alpha chain | *C. subellipsoidea* | XP_005651460 | | 98 | | |
| *aTUB-2* | MMETSP0491_2-20121128\|15061_1 | Tubulin alpha chain | *C. subellipsoidea* | XP_005651460 | | 98 | | |
| *bTUB* | MMETSP0491_2-20121128\|8818_1 | Tubulin beta chain | *M. neglectum* | XP_013901548 | | 98 | | |
| *UBCE* | MMETSP0491_2-20121128\|9783_1 | Ubiquitin-conjugating enzyme | *C. subellipsoidea* | XP_005649772 | | 83 | | |
| *PGK* | MMETSP0491_2-20121128\|3830_1 | Phosphoglycerate kinase | *A. pyrenoidosa* | AKP17751 | | 71 | | |
| *RPS10* | MMETSP0491_2-20121128\|7667_1 | 40S ribosomal protein S10 | *K. nitens* | GAQ92829 | | 72 | | |
| *eIF2-1* | MMETSP0491_2-20121128\|1472_1 | Initiation factor 2 | *C. subellipsoidea* | XP_005643492 | | 61 | | |
| *eIF2-2* | MMETSP0491_2-20121128\|9725_1 | Translation initiation IF-2, mitochondrial | *N. sylvestris* | XP_009761958 | | 46 | | |
|  |  |  |  |  | |  | | |
| Gene symbol | Description | Accession No | *T. chui* strain |  | |  | | |
| *rbcL* | Chloroplast ribulose-1,5-bisphosphate carboxylase/oxygenase large subunit | HF931099 | ICMAN_CSIC |  | |  | | |
| *18S* | 18S rRNA gene | HF931098 | ICMAN_CSIC |  | |  | | |
|  |  |  |  |  | |  | | |
| TARGET GENES | | | | |  |  |  |  |
| Gene symbol | Sequence ID (*T. chui* PLY429) | Best hit (BLASTp) | Species | Accession No | | Identity (%) | |  |
| *AGPL* | MMETSP0491_2-20121128\|23823_1 | Glucose-1-phosphate adenylyltransferase large subunit 1, chloroplastic isoform X4 | *A. officinalis* | XP_020258103 | | 59 |  |  |
| *AGPS* | MMETSP0491_2-20121128\|4141_1 | ADP-glucose pyrophosphorylase small subunit | *D. parva* | AEL29992 | | 66 |  |  |
