# Supplementary material for: Selection and validation of reference genes for quantitative real-time PCR in the green microalgae Tetraselmis chui
Source: PLoS One. 2021 Jan 14;16(1):e0245495. doi: 10.1371/journal.pone.0245495 (PMC7808622; doi:10.1371/journal.pone.0245495)
Supplement: S1 Raw images — (PDF) [file pone.0245495.s010.pdf]

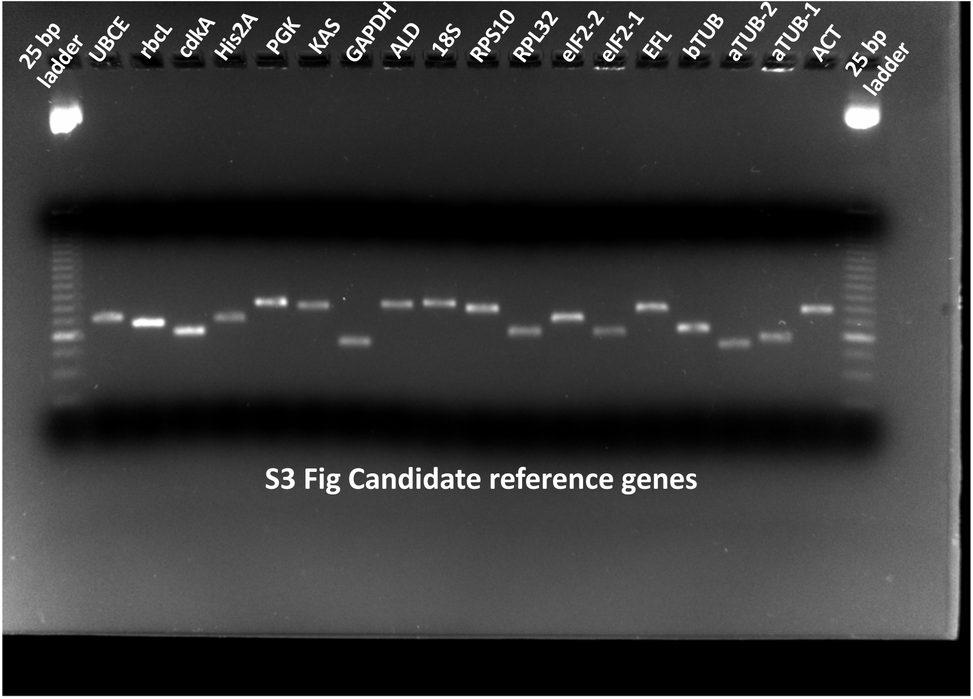

S3 Fig Candidate reference genes

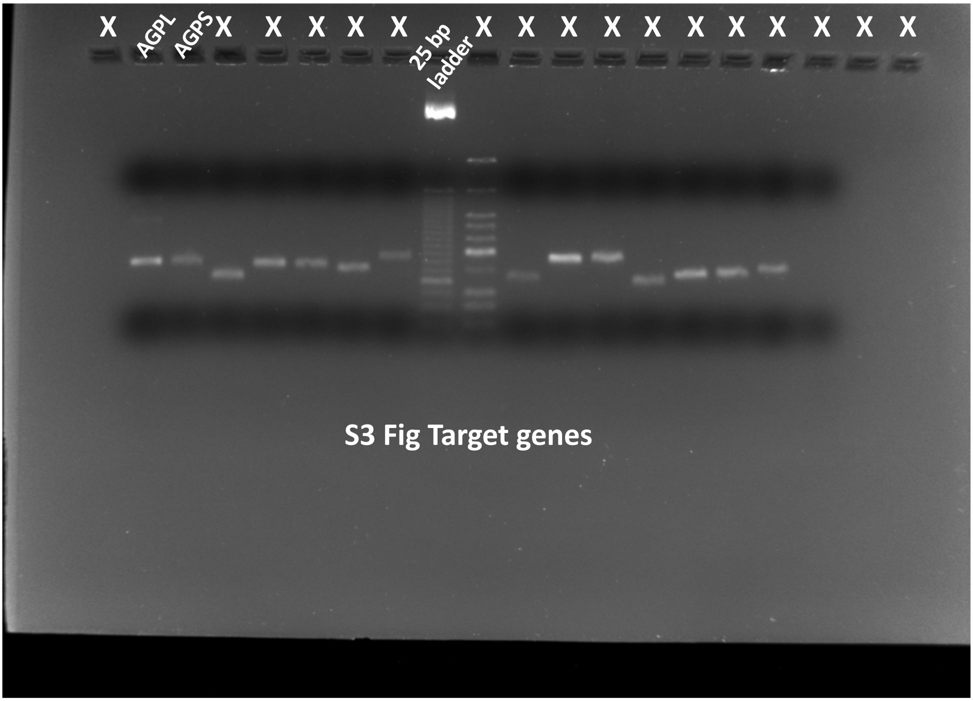

S3 Fig Target genes
